# A simple method to isolate structurally and chemically intact brain vascular basement membrane for neural regeneration following traumatic brain injury

**DOI:** 10.1186/s40824-023-00341-6

**Published:** 2023-01-12

**Authors:** Wanqing Ji, Zhiru Wu, Jiaming Wen, Hengxin Tang, Zhuopeng Chen, Bo Xue, Zhenming Tian, Yueyang Ba, Ning Zhang, Xuejun Wen, Bo Hou

**Affiliations:** 1 Department of Obstetrics, Guangzhou Women and Children’s Medical Center, Guangzhou Medical University, Guangdong Provincial Clinical Research Center for Child Health, Guangzhou, 510623 China; 2 Department of Nephrology, Dongcheng branch of the First Affiliated Hospital of Anhui Medical University, Hefei, China; 3 Guangzhou First People’s Hospital, South China University of Technology, Guangzhou, China; 4 Department of Neurosurgery, The Third Affiliated Hospital, Sun Yat-sen University, Guangzhou, 510630 Guangdong Province China; 5 Shared Research Facilities, West Virginia University, 1306 Evansdale Drive, Morgantown, WV 26506 USA; 6 Department of Biomedical Engineering, Institute For Engineering and Medicine, Virginia Commonwealth University, Room 399, 601 West Main Street, Richmond, VA 23220 USA; 7 Department of Chemical and Life Science Engineering, Virginia Commonwealth University, 601 West Main Street, Richmond, VA 23220 USA

**Keywords:** Vascular basement membrane, Microvascular scaffold, Brain extracellular matrix, Biomaterials, Traumatic brain injury

## Abstract

**Background:**

The brain vascular basement membrane (brain-VBM) is an important component of the brain extracellular matrix, and the three-dimensional structure of the cerebrovascular network nested with many cell-adhesive proteins may provide guidance for brain tissue regeneration. However, the potential of ability of brain-VBM to promote neural tissue regeneration has not been examined due to the technical difficulty of isolating intact brain-VBM.

**Methods:**

The present study developed a simple, effective method to isolate structurally and compositionally intact brain-VBM. Structural and component properties of the brain-VBM were characterized to confirm the technique. Seed cells were cocultured with brain-VBM in vitro to analyze biocompatibility and neurite extension. An experimental rat model of focal traumatic brain injury (TBI) induced by controlled cortical impact were conducted to further test the tissue regeneration ability of brain-VBM.

**Results:**

Brain-VBM isolated using genipin showed significantly improved mechanical properties, was easy to handle, supported high cell viability, exhibited strong cell adhesive properties, and promoted neurite extension and outgrowth. Further testing of the isolated brain-VBM transplanted at lesion sites in an experimental rat model of focal TBI demonstrated considerable promise for reconstructing a complete blood vessel network that filled in the lesion cavity and promoting repopulation of neural progenitor cells and neurons.

**Conclusion:**

The technique allows isolation of intact brain-VBM as a 3D microvascular scaffold to support brain tissue regeneration following TBI and shows considerable promise for the production of naturally-derived biomaterials for neural tissue engineering.

**Graphical Abstract:**

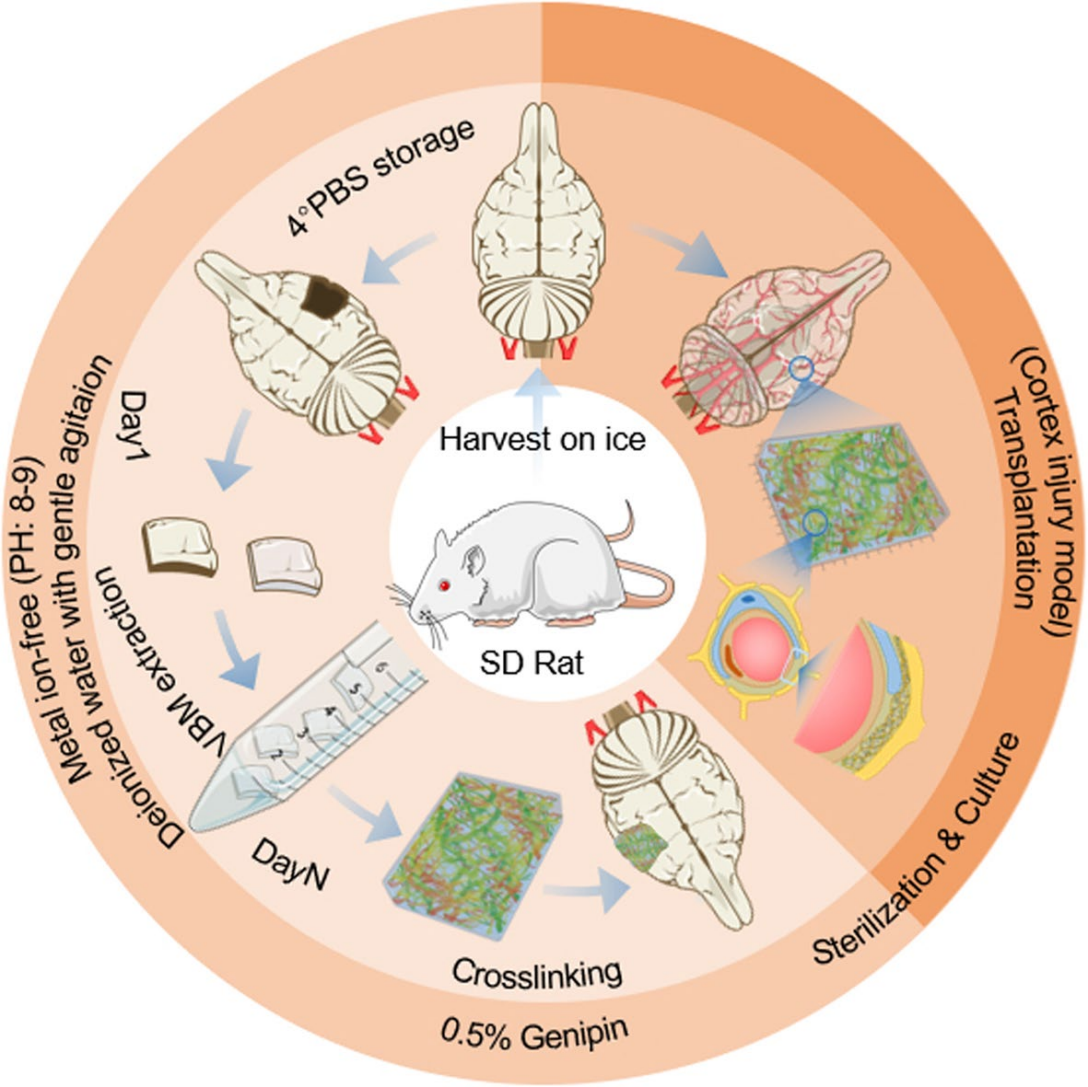

**Supplementary Information:**

The online version contains supplementary material available at 10.1186/s40824-023-00341-6.

## Introduction

Brain injury caused by mechanical or pathological damage often results in the lack of tissue structural integrity, which worsens because of the consequential impairment caused by an ischemic microenvironment and inflammation-related factors [1, 2]. Although some indications of intrinsic neurogenesis in the central nervous system (CNS) have been reported, the inability of endogenous neural progenitor cells (NPCs) to infiltrate and repopulate a lesion site due to the absence of structural support often impedes the regeneration of brain tissue [3].

Despite an inhibitory environment at the lesion site, some proof-of-principle studies have confirmed that biomaterials can at least partly promote nerve regeneration at lesion sites in the CNS [4, 5]. Biomaterials may confer cell-adhesive properties, promote cell proliferation, migration, and differentiation [6], and induce revascularization at the tissue defect site. Further, biomaterials may serve as carriers for the controlled release of therapeutic agents to reduce tissue inflammation and scarring, all of which act in concert to promote neural tissue regeneration. Ideally, engineering scaffolds should mimic as many features of the native brain extracellular matrix (ECM) as possible.

Common biomaterials (supporting Table 1) used to facilitate brain tissue regeneration are biopolymers in the forms of porous meshwork, filaments (electrospinning), and hydrogel [7]. Some bioactive cues are incorporated into these materials to increase survival, proliferation, differentiation, or maturation of NPCs [8, 9]. Obviously, these engineered scaffolds differ significantly from the real brain-ECM in their physical structure and their composition. These great differences often cause inflammation and damage to brain tissue [10]. Ideal alternative brain biomaterials should be characterized by a high degree of specific homology with the components and structure of the ECM within the brain.

The brain ECM consists of three major regions: the vascular basement membrane (VBM), the perineural net, and the neural interstitial matrix [11]; the latter two regions are discretely distributed throughout the brain parenchyma without continuous structures. In contrast, brain-VBM originates from blood vessels and is characterized by the three-dimensional (3D) structure of the cerebrovascular network. However, isolation of structurally intact brain-VBM is not easy. In fact, attempts to isolate brain-VBM began many years ago [12, 13]. Relevant studies have been based on the exploration of internal mechanistic questions such as blood–brain barrier formation and function [14], cerebral endothelial cell biology, and vascular invasion of intracranial malignant tumors [15]. In these studies, many techniques have been used to isolate brain microvessels. However, these methods yield only VBM fragments rather than structurally intact VBM. Another major problem common to all these techniques is the low purity and homogeneity of the acquired blood vessels, which inevitably limits the reproducibility and accuracy of subsequent experiments. Despite the scientific community’s awareness of the value of structurally intact brain-VBM as a biological material [16, 17], a technique to isolate structurally and compositionally intact brain-VBM does not exist, which hinders the investigation of the biomedical potential of brain-VBM.

The present study sought to develop a technique to isolate intact brain-VBM from native brain tissue; to investigate the structural and compositional characteristics and cytocompatibility of brain-VBM and its biological effects on cells; and to evaluate the efficiency of intact brain-VBM as a scaffold biomaterial in improving neural regeneration in an experimental rat model of traumatic brain injury (TBI).

## Results

### Brain-VBM preparation and crosslinking

**Fig. 1:**
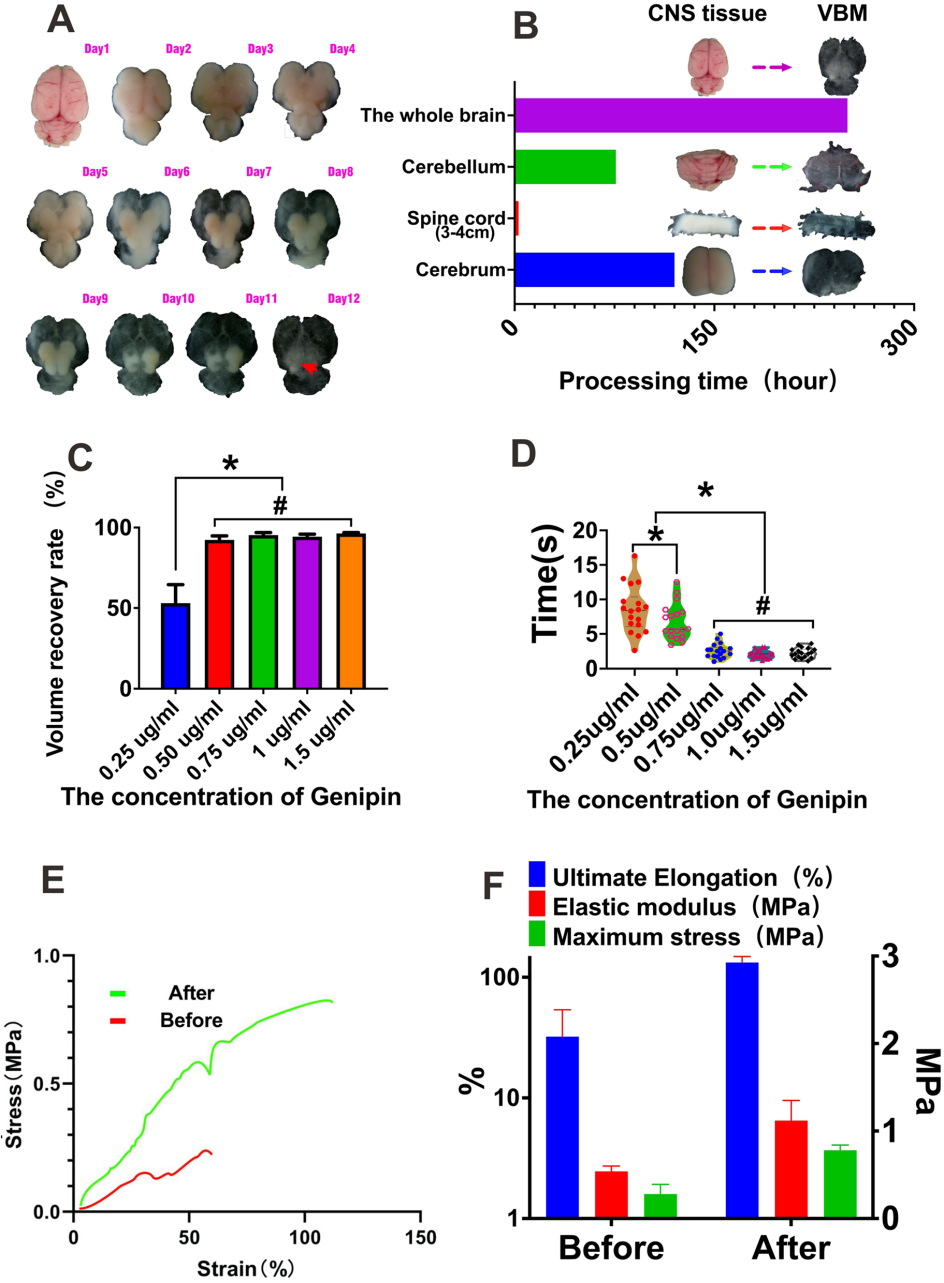
Preparation and crosslinking of brain-VBM: **A** Schematic showing the design of this study, including brain-VBM preparation and processing, cell compatibility testing, and repair of the animal cortex after injury. **A** and **B** Dynamic display of the process of preparing brain-VBM of the entire brain (arrow indicates the thicker tissue, but not the remaining brain parenchymal tissue). The time required for preparing VBM from the central nervous system of different sizes and tissue sources varied. **C-E** show significant improvement in the mechanical properties of the brain-VBM after genipin crosslinking: Brain-VBM had significant shape memory characteristics. The concentration of the crosslinking agent affected the brain-VBM volume recovery rate (**C**) and time required for shape recovery (**F**). Statistical significance was set at *P* < 0.05; *P < 0.05. # indicates nonsignificance

The inherent conditions underlying the brain’s lack of elasticity and fragile texture render conventional decellularization technologies unsuitable for preparing structurally intact brain-VBM. We found that the structurally intact brain-VBM could be easily separated by using weakly alkaline double-distilled water (pH = 8–8.5) combined with slight mechanical agitation (the preparation timeline is shown in Fig. 1), and the process was simple and efficient. Isolation of the intact brain-VBM using this method only required approximately 12 days (Fig. 1B). Under the condition of continuous mechanical agitation, the brain tissue gradually became transparent from the outside to the inside starting on day 3. By frequently replenishing the liquid with fresh alkaline double-distilled water, the entire cortex and cerebellum of the brain became completely transparent on day 10 (see Fig. S1 A), and the remaining regions (corpus callosum, thalamus, and diencephalon) were completely transparent on day 12 (from a superior view; the area indicated by the arrow was opaque due to the thick tissue with overlapping structures in Fig. 1B). This method also works for other tissues in the CNS (Fig. 1C), although treatment times vary significantly. The separated VBM could maintain the overall anatomical shape and volume of the original tissue, but the weight (dry weight) was only 0.01% of that of the original tissue. Fresh brain-VBM has very poor mechanical properties. It shrinks rapidly during dehydration, and without fluid support, returning to its original size is difficult.

To improve mechanical properties (for subsequent applications, including 3D coculture of composite cells and in vivo transplantation), we crosslinked the isolated brain-VBM with genipin. The protein components of the brain-VBM exhibited a uniformly light blue color after crosslinking with genipin (see Fig. S1 G). The mechanical properties of brain-VBM changed dramatically after crosslinking. Mechanical analysis revealed that, after crosslinking, the stiffness (elastic modulus; before 0.54 ± 0.06 MPa vs after 1.12 ± 0.23 MPa; *P* < 0.01), strength (maximum stress; before 0.28 ± 0.11 MPa vs after 0.78 ± 0.06 MPa; *P* < 0.05), and elasticity (ultimate elongation of specimens; before 32.2 ± 21.6% vs after 132.5 ± 16.1%; *P* < 0.05) of the brain-VBM improved significantly. Crosslinked brain-VBM (dehydrated state) had its original volume, structure, and morphology restored to varying extents within a few seconds after hydration swelling. The volume recovery ratio was only 50% if the concentration of genipin was 0.25 mg/mL. If the genipin concentration for crosslinking was ≥0.5 mg/mL, the volume recovery ratio was 97.4–99% (*P* > 0.05). In the absence of crosslinking, the volume recovery ratio of brain-VBM was only 5%. Additionally, the impact of recovery time (RT) on the shape memory function was studied, which had a certain correlation with the genipin concentration. After crosslinking at the concentration of 0.25% (w/v), the RT required for complete expansion of brain-VBM was 8.32 ± 6.1 s, and at the concentration of 0.75% (w/v), the RT was significantly shortened to 3.34 ± 0.79 s (*P* < 0.001). Further, increasing the genipin concentration (with the same crosslinking duration) did not significantly affect the RT (*P* > 0.05).

### Characteristics of brain-VBM

#### Morphological and structural analysis

**Fig. 2:**
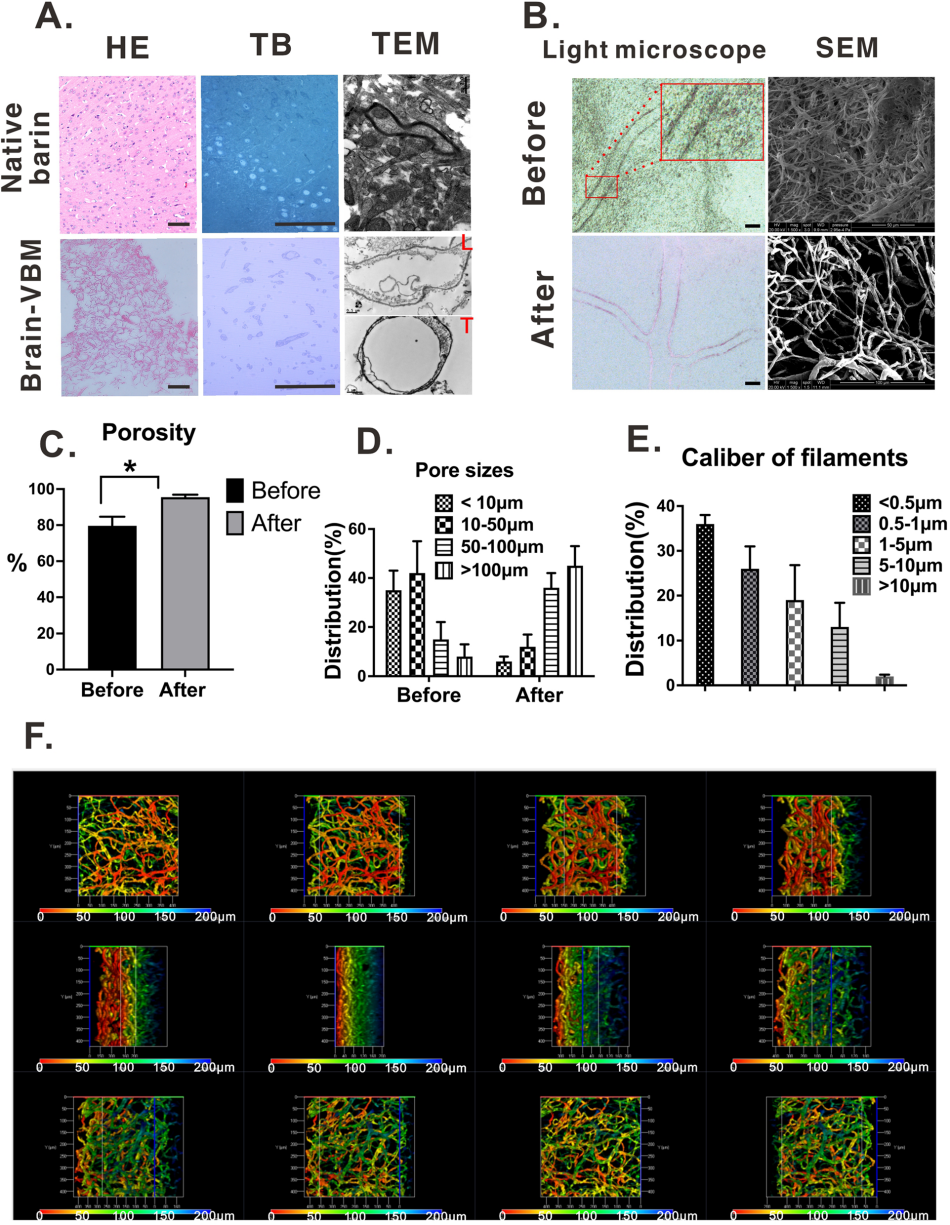
Characteristics of the brain-VBM structure. **A** Compared with normal brain tissue (**A**. 1–3), the structure of brain-VBM was loose. Hematoxylin and toluidine blue staining indicated that the cellular components of brain-VBM were completely removed. TEM revealed that brain-VBM was composed of interlinked hollow basement membrane tubes (L: longitudinal view; T: transverse view). **B**-**D** Light microscopy and SEM showed that the porosity of brain-VBM was significantly increased after crosslinking, with an intersecting branch-like structure (**E** shows the distribution of different calibers of VBM). The 3D reconstruction (**F**) of the confocal scanning results (see video 1) of laminin-stained brain-VBM samples shows multiple views of the structure and morphology of brain-VBMs. The depth scale in **F** shows different colors according to the z-positions. Statistical significance was set at *P* < 0.05; *P < 0.05

Light microscopy, confocal fluorescence microscopy (3D reconstruction after laminin staining), and scanning electron microscopy (SEM) of the translucent brain-VBM (Fig. 2A, B, F) indicated that brain-VBM had a network structure composed of many vessel-like and intricately intersecting filaments with varying diameters. The diameters of the filaments were generally 0.5–5 μm. Hematoxylin–eosin (H&E) staining revealed that the structure of brain-VBM was loose and porous, and no nuclei were observed under the microscope. Before crosslinking, the diameters of over 70% of the pores were < 50 μm, and the porosity was 79.6 ± 5.1%; after crosslinking, the porosity increased to 95.5 ± 1.4% (*P* < 0.01; vs before), and the diameters of over 75% of the pores were > 50 μm. Toluidine blue (TB) staining (Fig. 2A) showed that the dark-stained myelin structure found in the native brain tissue had disappeared completely. TEM further confirmed that the branch- and trunk-like structures were hollow (transverse (T) and longitudinal (L) views) and that the walls were composed of structurally intact basement membranes. The axons, myelin, and all cellular components found in the native brain tissue had completely disappeared. These results indicate that the processed brain-VBM had a complete hollow tube-like microvascular spatial structural network (see video 1) and that the cellular components, including nuclei, myelin, and axons, were removed during processing.

#### Component analysis

**Fig. 3:**
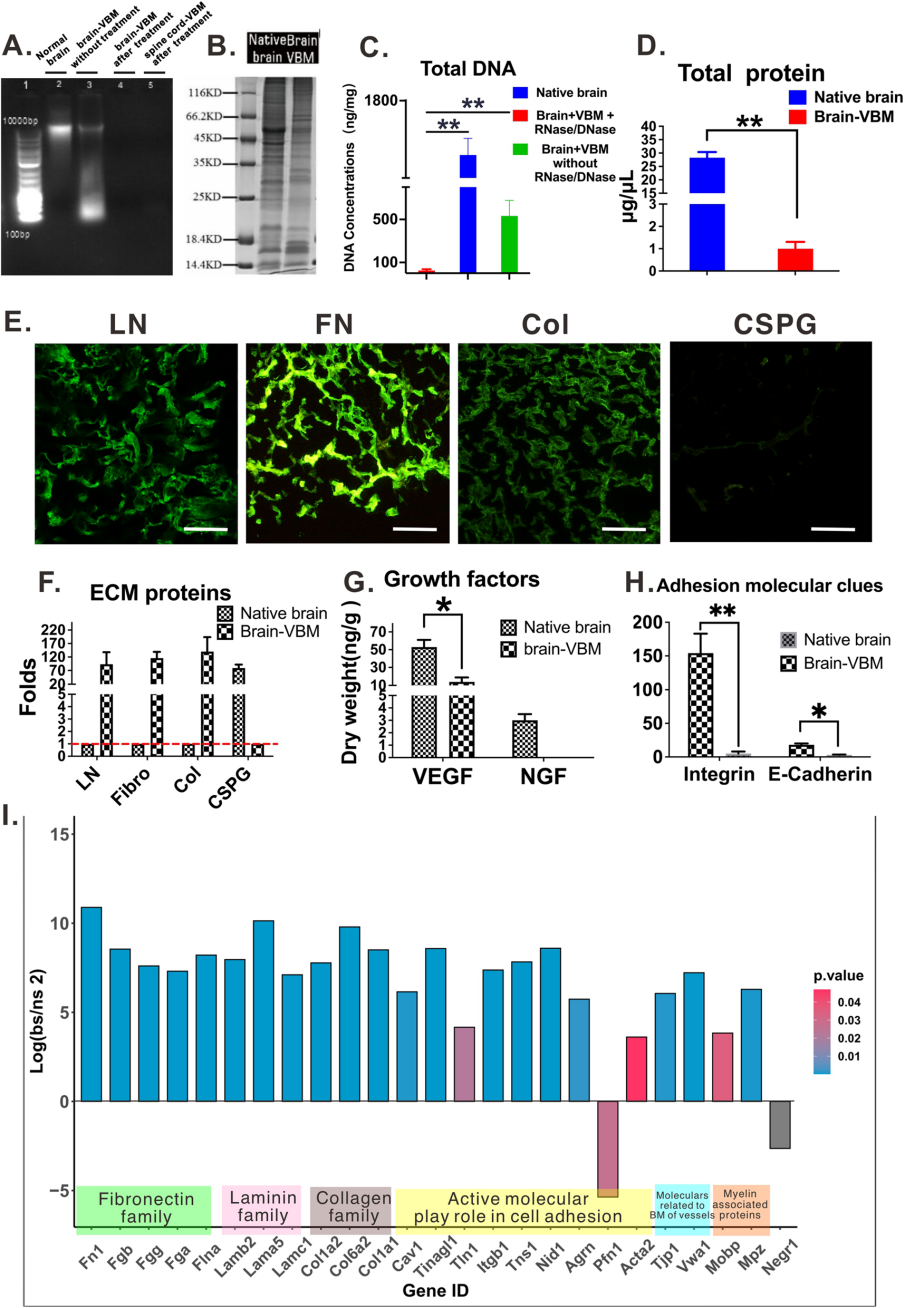
Characteristics of brain-VBM components. **A**-**D** Comparisons of the proteins and DNA in the native brain tissue and in the brain-VBM. **E**-**F** show that brain-VBM contained more ECM-promoting factors, including laminin, fibronectin, and collagen, but very few inhibitory components such as chondroitin sulfate proteoglycan. In terms of active growth factors and adhesive molecules, compared with normal brain tissue, brain-VBM still had a considerable amount of vascular endothelial growth factor (**G**) and most known adhesive molecular factors (**H**), but nerve growth factor was almost undetectable (**G**). **I** shows 25 significantly differentially expressed proteins related to ECM and the ratio of their respective contents in brain-VBM to native brain. Statistical significance was set at *P* < 0.05; *P < 0.05, **P < 0.01

Component analysis further confirmed the origin of brain-VBM. DNA electrophoresis (Fig. 3A, C) revealed that brain-VBM (lane 2) treated as described in section 1 still contained a substantial amount of DNA (532 ± 145 ng per mg dry weight). After DNase/RNase treatment, the residual DNA in VBM-1 (lane 4; brain-VBM) and VBM-2 (lane 5; VBM from spinal cord) was < 100 bp. Compared with that in the native brain (lane 3), quantification of dsDNA using Tissue DNA Kits indicated that brain-ECM retained < 25 ± 13 ng dsDNA per mg dry weight. Compared to observations in native brain tissue (1589 ± 65 ng per mg dry weight), more than 99% of the DNA content of the native brain tissue was removed during processing (see Fig. S1 B).

Total protein analysis revealed that the total protein concentration of the native brain (Fig. 3D) was 28.291 μg/mL, while that of the brain-ECM was only 2.54 μg/mL (*P* < 0.001). SDS-PAGE imaging (Fig. 3B) showed the protein distribution of brain-ECM compared with native brain tissue. Immunofluorescence staining (Fig. 3E) and ELISA (Fig. 3F) revealed that brain-VBM contained abundant ECM proteins from the VBM, such as collagen, laminin (LN), and fibronectin (FN). The concentrations of these matrix proteins were generally higher than those in the native brain (fold = (brain-VBM)/native brain: collagen: 154 ± 54-fold; LN: 93 ± 47-fold; FN: 116 ± 27-fold). However, ECM coming from components of the brain parenchyma, such as chondroitin sulfate proteoglycans (CSPGs) in brain-VBM, was significantly reduced (by 79 ± 13-fold) when compared with that in native brain tissue. ELISA (Fig. 3H) showed enrichment of two types of cell adhesion molecules, i.e., integrin (calcium-independent) and cadherin (calcium-dependent), in brain-VBM (154 ± 29 pg/mL and 18 ± 2 pg/mL, respectively) compared to native brain tissue (5 ± 3 pg/mL and 3 ± 0.6 pg/mL, respectively). Brain-VBM retained detectable concentrations of some soluble growth factors (Fig. 3G), such as vascular endothelial growth factor (VEGF) at 34% (14 ± 5 ng/g in brain-VBM vs 53 ± 8 ng/g in native brain tissue). However, nerve growth factor was largely washed out during processing (> 95%; almost undetectable in brain-VBM vs 3 ± 0.5 ng/g in native brain tissue).

Proteomic analysis (Fig. 3I and Fig. S3) was conducted to examine the protein components of brain-VBM. When comparing brain-VBM with native brain, among the top 50 upregulated proteins in brain-VBM, 19 (38%) were ECM proteins, and among the top 50 downregulated proteins, only one (2%) was an ECM protein. Gene ontology (GO) analysis of the differentially expressed proteins between native brain tissue and brain-VBM indicated that among the top 20 functional characteristics, 17 were related to ECM and cell adhesion, five of eight (62.5%) GO terms fell under biological processes, six of six (100%) GO terms fell under molecular functions, and six of six (100%) GO terms fell under cellular component.

Among the top 20 most enriched KEGG pathways, four contained the most differentially expressed proteins, and three pathways were related to ECM and adhesion factors. The focal adhesion and ECM–receptor interaction pathways promote growth cone guidance, cytoskeleton regulation, remyelination, and synaptogenesis. The PI3K/Akt and MAPK signaling pathways activate local receptors to promote axon growth and axon attraction, and they may be activated during axon chemotactic growth. Figure S3 A shows the heatmap of the 100 most differentially expressed proteins (differentially expressed proteins were screened by > 2-fold upregulation or < 0.5-fold downregulation with *P* < 0.05; a total of 100 proteins were identified, including 51 upregulated proteins and 49 downregulated proteins). We selected 25 proteins that were related to brain-ECM. Figure 3I shows the proportional concentrations of these proteins in brain-VBM and native brain tissue (their log_2_ relationships). The results indicated that ECM proteins such as FN, LN, and collagen were generally upregulated 100–2000-fold in brain-VBM relative to those in the native brain. In summary, our results demonstrated that the produced brain-VBM preserved ECM proteins from the VBM but lost ECM proteins from the brain parenchyma during processing.

### Cytocompatibility of brain-VBM

**Fig. 4:**
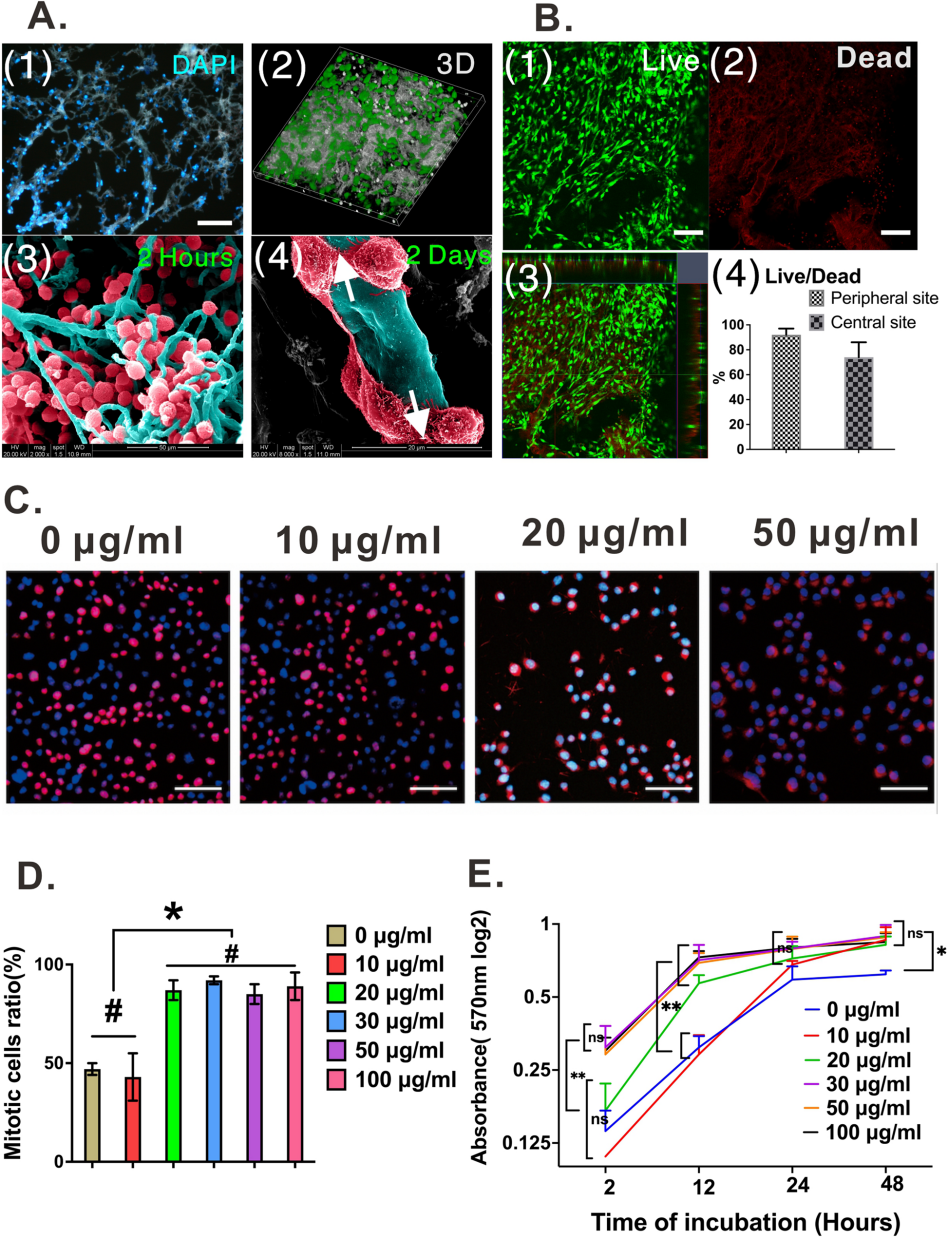
Cytocompatibility of brain-VBM. **A** (1) and **A** (3) show that after 2 h of coculture with brain-VBM, the cells evenly attached to the surfaces of the VBM scaffolds. After coculture for 48 h, 3D reconstruction of confocal imaging indicated that cells were evenly distributed on brain-VBM, and the structure of brain-VBM was not shrunken. Cells migrated to the surface of the BM branches. Cell–cell contacts (white arrows) are shown in **A** (4). **B** Live/dead assay suggested growth of cells on brain-VBM. **C-E** MTT and EdU assays revealed that brain-VBM components (even at high concentrations) did not hinder cell proliferation; in contrast, they promoted cell proliferation at certain concentrations (> 20 μg/mL). Statistical significance was set at *P* < 0.05; *P < 0.05, **P < 0.01, and ns and # indicate nonsignificant

Cytocompatibility is an important parameter to assess whether brain-VBM can be transplanted in vivo and used as a cell carrier. After coculturing bone marrow–derived mesenchymal stem cells (BMSCs) with brain-VBM for 2 h, light microscopy (see Fig. S1 C) and SEM (Fig. 4A (3)) revealed many BMSCs adhered to the surface of the brain-VBM without spreading. After 48 h, SEM (Fig. 4A(4)) images indicated that the BMSCs had extended and migrated into brain-VBM, and the cells were connected to each other with many membranous protrusions (white arrows), suggesting that brain-VBM could provide BMSCs with sites and space for adhesion and growth. After 2 days of coculture, live/dead staining (Fig. 4A (2), B (1–4)) followed by confocal fluorescence imaging showed that the survival rate of BMSCs at the peripheral zone of brain-VBM was > 92%, and the rate was > 74% at the center (see Fig. S2), indicating that brain-VBM could provide a suitable internal microenvironment for BMSC survival. MTT/CCK8 toxicity experiments revealed (Fig. 4C-E) that when the brain-VBM concentration was higher than 20 μg/mL, brain-VBM components significantly promoted cell proliferation. Examination of cell mitosis by EdU showed the same. In the brain-VBM-negative group and the 10 μg/mL group, the proportions of mitotic cells were 56 ± 7% and 58 ± 5%, respectively. When the concentration of brain-VBM was higher than 20 μg/mL, mitotic cells exceeded 95% of the total cells, suggesting that brain-VBM significantly promoted cell proliferation.

**Fig. 5:**
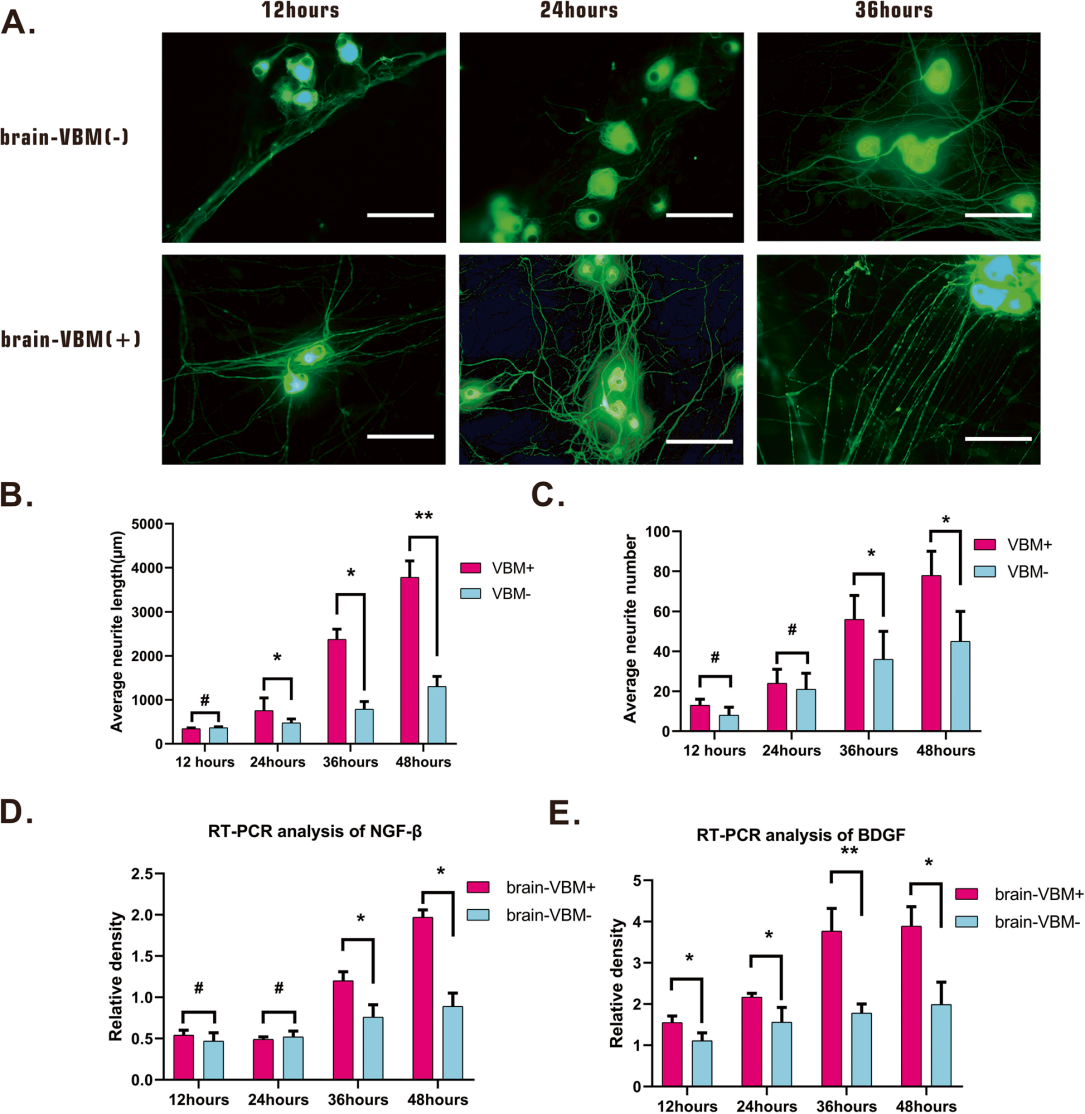
Effect of brain-VBM on neuron and neurite extension. NF-200 staining shows the morphology of cortical neurons with or without brain-VBM. After 36 h of coculture, the components of brain-VBM had marked positive effects on the neurite length (**B**) and axon number (**C**) of cerebral cortical neurons. **D**, **E** NGF-β and BDGF mRNA expression of neurons under different culture conditions. Bar =100 μm. Statistical significance was set at *P* < 0.05; *P < 0.05, and # indicates nonsignificant

Cortical neurons were used to examine the effects of brain-VBM on axon outgrowth (Fig. 5A). We found that brain-VBM at 50 μg/mL significantly affected the number and length of regenerated axons. The average neurite length in the brain-VBM-group was almost two and three times that in the control group at 36 and 48 h, respectively. The average neurite number in the brain-VBM group was significantly higher than that in the control group (56 ± 12 per neuron vs 36 ± 14 per neuron at 36 h; 78 ± 12 vs 45 ± 15 per neuron at 48 h). In addition, the measurement results of NGF-β and BDGF expression further confirmed the effect of brain-VBM on neurons. Specifically, brain-VBM promoted BDGF expression (Fig. 5D, *P* < 0.05) but had little effect on NGF-β expression in cortical neurons at the early stage (< 24 h) of coculture and significantly affected both BDGF expression and NGF-β expression in neurons after 24 h of coculture (Fig. 5D, E). These results suggest that the components of brain-VBM promote axon regeneration.

### Postoperative nerve tissue regeneration

**Fig. 6:**
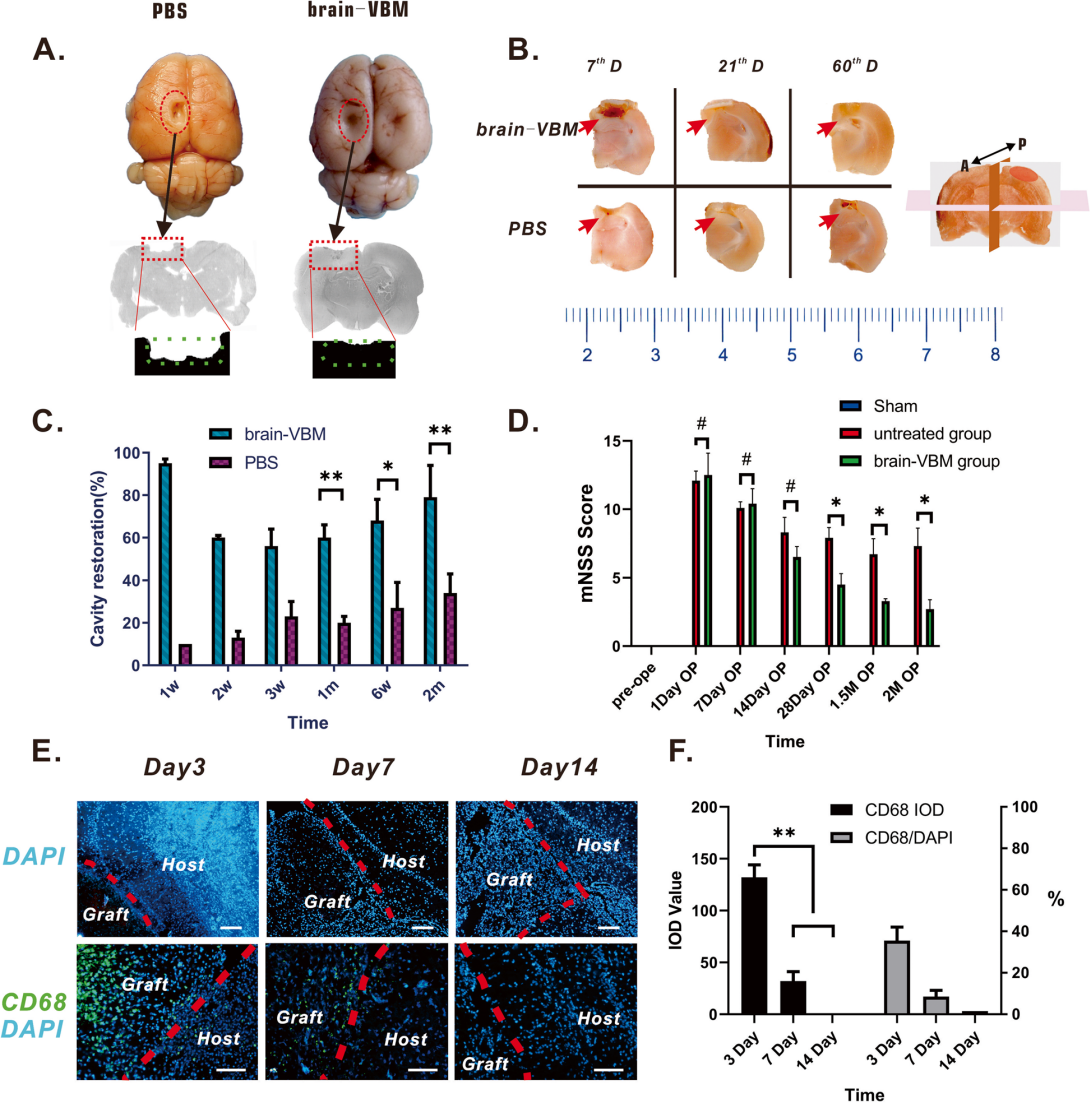
Tissue restoration after brain-VBM repair. **A** Comparison of the overall view and H&E-stained lesion region at 2 months after surgery between the brain-VBM group and the PBS group. The green dashed box indicates the scope of the original lesion site. The repair status of the lesion cavity in the two groups (**B**, red arrows; A: anterior, P: posterior) at different time points and the quantitative comparison (**C**) showed that cavity restoration in the brain-VBM group was significantly better than that in the PBS group. **D** In terms of neurological functional recovery, modified neurological severity scores (mNSSs) in the brain-VBM group at 4, 6, and 8 weeks after surgery were significantly lower than those in the PBS group at the same time points. **E** shows cell invasion (DAPI in graft site) and immune reactivity (CD68 in graft site) in the lesion cavity region in the brain-VBM group at 3 days, 7 days, and 14 days after surgery. **F** Quantification analysis indicates that the inflammation response was significantly reduced at postoperative day 7 and completely disappeared at postoperative day 14. Statistical significance was set at *P* < 0.05; *P < 0.05, **P < 0.01, and # indicates nonsignificant

Local brain tissue regeneration (based on H&E staining) after transplantation showed that local brain tissue regeneration was significantly better in the brain-VBM group than in the PBS group between 1 and 2 months after transplantation (Fig. 6A). The tissue regeneration areas of the lesion region in the two groups were 60.3 ± 6.1% and 20.2 ± 3.4% at 4 weeks, 68.6 ± 10.2% and 27.6 ± 12.1% at 6 weeks, and 79.9 ± 15.3% and 34.7 ± 9.5% at 8 weeks, respectively (Fig. 6B-C). The modified neurological severity score (mNSS) (Fig. 6D), one of the most common neurological scales used in animal studies, peaked on postoperative day 1 in both groups, and neurological functional recovery in the two groups did not differ significantly within 2 weeks after surgery. At 4 weeks, the mNSS of the brain-VBM group was significantly lower than that of the PBS group, which difference become more pronounced at 6 weeks and 8 weeks after surgery. DAPI staining (Fig. 6E,F) of the brain-VBM transplantation group indicated that at postoperative day 3, some cells had grown into the brain-VBM, more than 40% of which were inflammatory responsive cells (CD68^+^ macrophages). At postoperative day 7, the number of cells growing into the brain-VBM further increased, with fewer than 10% being CD68^+^ macrophages. At postoperative day 14, the lesion site was filled with cells other than CD68^+^ macrophages. These findings suggest that brain-VBM can be used as a scaffold or injectable hydrogel to fill in the defect caused by a brain tissue lesion and can also promote the recovery of neurological function to some extent. The local inflammatory response at the lesion site ended within 2 weeks after local brain-VBM transplantation.

**Fig. 7:**
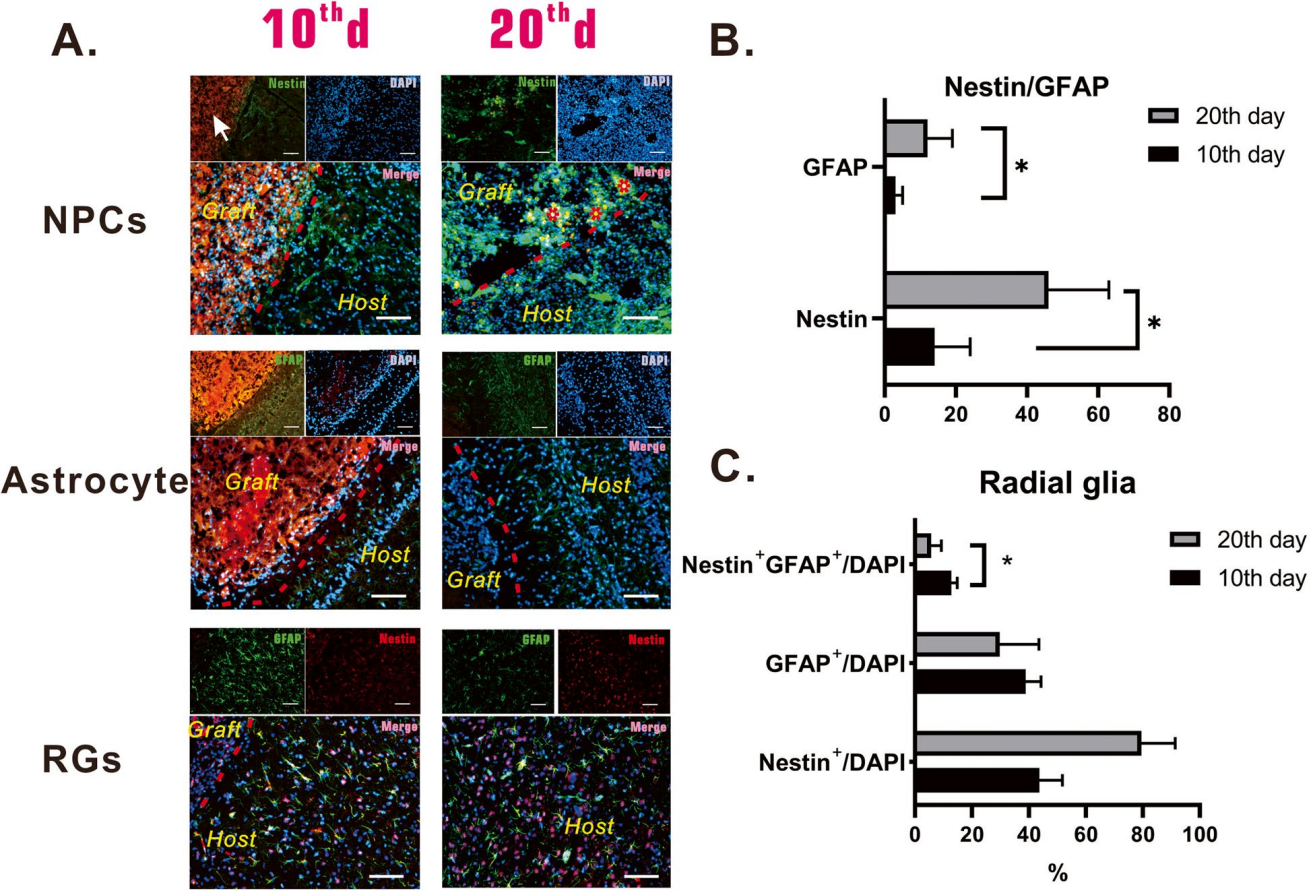
Glial cells and neural progenitor cells populated lesion sites 10 and 20 days after brain-VBM placement. Ten days after surgery, some nestin-expressing cells (NPCs) were identified at the perimeter of the brain-VBM, and nestin expression further increased at postoperative day 20. At 10 days, many astrocytes surrounding the lesion site were activated, but few astrocytes had migrated into the brain-VBM; at day 20, although more astrocytes were activated, only a few GFAP-expressing cells were present in the lesion site. Radial glial progenitor cells (RGPs) are NPCs. At the early postoperative stage, we found that RGPs were present around the lesion site, but they were less at day 20. Red dashed lines are the boundary between lesion site and host tissue. Triangles (red asterisks) represent a completely decomposed brain-VBM. Scale bars: 100 μm. Statistical significance was set at *P* < 0.05; *P < 0.05

**Fig. 8:**
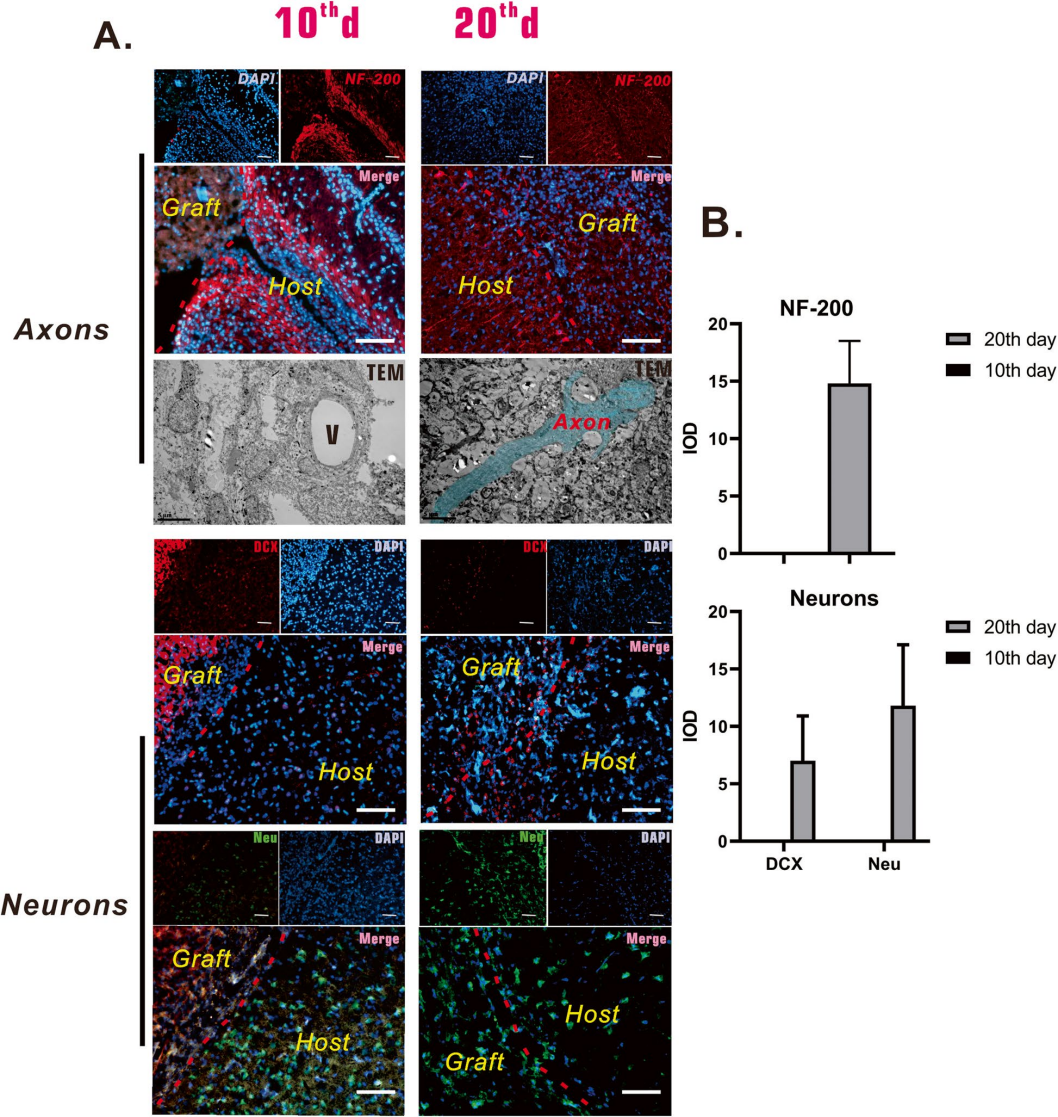
Axonal (NF-200 staining and TEM observation) and neuronal (DCX for immature neurons and NeuN for mature neurons) regeneration in lesion and peri-lesion regions 10 days and 20 days after brain-VBM placement. At 10 days, few of axons had crossed the injury boundary, and some vessels were found in the lesion area. Almost no neurons had invaded the lesion site, and a substantial number of axons and neurons were present in the graft area. At 20 days, neurons had invaded. Red dashed lines show the boundary between lesion site and host tissue. Arrowheads represent brain-VBM (with spontaneous fluorescence). V indicates vessels. Scale bars: 100 μm

Immunohistochemistry of the local regenerated tissue was performed to confirm neural tissue regeneration and to explore the role of brain-VBM in the process. Early results (at postoperative day 10) indicated that in the presence of brain-VBM, many cells migrated into the brain-VBM scaffold (DAPI results). At this time, the brain-VBM (Fig. 7A, orange area indicated by the white arrowhead) was still in the porous 3D structure. Many cells surrounding the lesion site expressed nestin, and some cells inside the lesion site also expressed nestin. At this time, activated glial cells (GFAP^+^) had mainly accumulated around the injured region, with only a limited number of GFAP^+^-positive cells inside the lesion site. GFAP/nestin double staining (Fig. 7C) revealed that 43% of the activated glial cells close to the injured region in the early stage also expressed nestin. At this time, axons (NF-200^+^) at the edge of the lesion site grew irregularly (Fig. 8A; Fig. S1 E), with no obvious axons entering the lesion site through the edge of the lesion. NPCs had formed in the dentate gyrus (subgranular zone, SGZ), with a significant trend of migration to the lesion site (Fig. 8A, Fig. S1 D). Immature neurons (DCX; Fig. 8A) still could not cross the injury boundary, and the mature neurons (Neu^+^) of the host tissue around the lesion site remained around the lesion site.

At 2 months after surgery, brain-VBM in the injured region was mostly degraded (Fig. 7A; red asterisks). Compared with observations at earlier time points, the injured region at this stage showed more nestin^+^ cells. Although many activated glial cells (GFAP^+^) surrounded the lesion site, the GFAP^+^ cells inside the injured region were not significantly elevated compared with observations at the early stage (Fig. S1 F). GFAP^+^ nestin^+^ radial glial cells (also referred to as radial glial progenitor cells, RGPs) around the injured region accounted for only 13% of GFAP^+^ cells (Fig. 7C), which was significantly lower than the percentage in the early stage (*P* < 0.01). Along with the gradual degradation of brain-VBM in the lesion site, some axons (NF-200 staining and TEM) crossed the lesion boundary (Fig. 8A). Compared with the normal axons in the surrounding host tissue, the distribution of these axons was disorderly. Neuronal staining (Fig. 8A) revealed more immature neurons (DCX^+^) and mature neurons (Neu^+^) appearing in and around the lesion at this stage.

### Brain-VBM scaffolds induced axon outgrowth and blood vessel regeneration in vivo

**Fig. 9:**
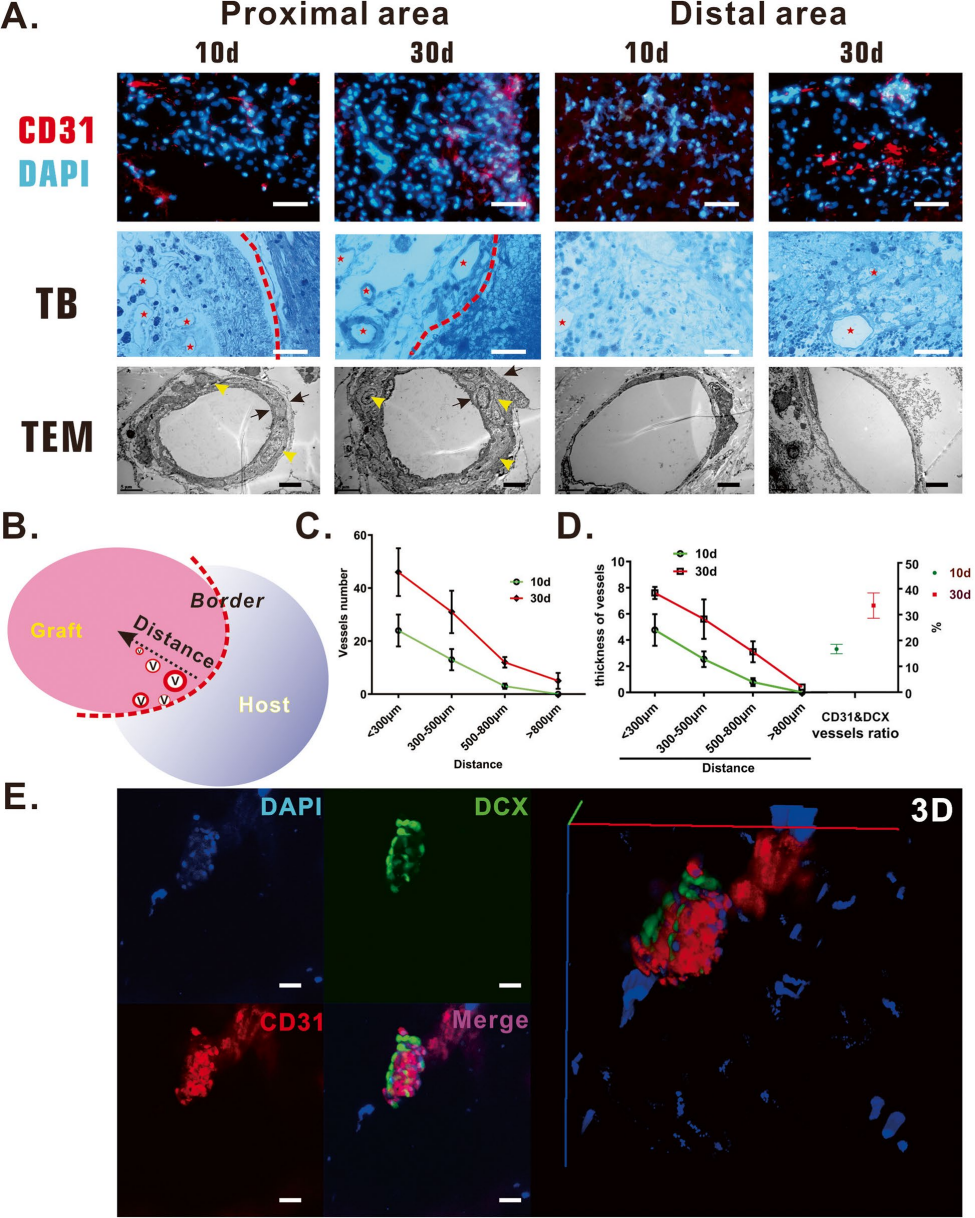
Local vascular induction after brain-VBM grafting. After surgery, CD31 immunostaining (**A**, endothelial cell marker) and toluidine blue staining showed angiogenesis at the proximal area (peripheral area surrounding the VBM grafts) even at 7 days after grafting, and more blood vessels were regenerated at proximal and distal areas. TEM indicated the luminal morphology of regenerated blood vessels at different time points and regions. The vessel wall at the distant regions was smooth and thin, while the wall of regenerated blood vessels near the lesion border was thick and wrapped with cells. **B**, **C**, and **D** show the relationship between the distance and blood vessel density and the relationship between the distance and the thickness of the blood vessels inside the lesion site. DCX and CD31 double immunostaining (**E**) revealed that inside the lesion site, the cells wrapping around the vascular wall (CD31^+^) were neurons (DCX^+^) in the early stage, with 17.6% of the vascular wall enveloped with DCX^+^ cells, which increased to 34.6% in the later stage). Red dashed lines are boundaries between lesion sites and host tissues

The above results confirmed that brain-VBM derived from cerebral blood vessels is enriched with VEGF. Therefore, we evaluated the effect of brain-VBM on local vascular regeneration. CD31 staining, toluidine blue staining, and TEM (Fig. 9A, C) revealed many new blood vessels at the borders of the grafting site 10 days after grafting (< 300 μm: 24.3 ± 5.6 vessels/10^4^ μm^2^; 300–500 μm: 13.4 ± 3.7 vessels/10^4^ μm^2^; and 500–800 μm: 3 ± 0.8 vessels/10^4^ μm^2^), and no obvious angiogenesis was noted in the center of the grafting sites. One month after grafting, 46 ± 9.3 vessels/10^4^ μm^2^ were identified at a distance < 200 μm from the border, 31.4 ± 7.8 vessels/10^4^ μm^2^ at a distance of 300–500 μm from the border, 12.8 ± 2.0 vessels/10^4^ μm^2^ at a distance of 500–800 μm from the border, and 4.8 ± 2.9 vessels/10^4^ μm^2^ at a distance > 800 μm from the border.

TEM (Fig. 9A) indicated more cells on the outer surface of the neovascular wall in the injured region, resulting in a thick vascular wall. At a distance < 200 μm from the border, the average thicknesses of vessel walls were 4.77 ± 1.21 μm at 10 days and 7.6 ± 0.46 μm at 30 days; in the 300–500-μm region, the values were 2.54 ± 0.6 μm and 5.6 ± 1.5 μm, respectively (Fig. 9D); and in the 500–800-μm area, the average thickness was 3.1 ± 0.8 μm at 30 days, which was much thicker than the value at 10 days (0.79 ± 0.3 μm). Within the central region of the lesion site (> 800 μm), the wall of the blood vessels was still thin even at 30 days after grafting. CD31 and DCX double staining (Fig. 9E) indicated that some of the cells wrapped around the vascular wall were neurons (DCX^+^). In the early stage after surgery, an average of 16.7% of newly formed vessels (CD31^+^) were wrapped by neurons (DCX^+^) inside the lesion sites, and this ratio increased to 33.5% (*P* < 0.05) at 30 days after surgery.

## Discussion

As a major part of the brain ECM, brain-VBM contains a unique native tissue structure and functional ECM components such as collagen, glycosaminoglycans (GAGs), and some growth factors. Under normal conditions, it presents a unique niche that directs cellular behavior in the brain (e.g., proliferation, migration, and differentiation), regulates angiogenesis, and maintains tissue homeostasis via mechanosensing and signaling. Brain-ECM also plays critical roles in the repair of injured brain tissue. Tissue regeneration after brain injury includes extremely complex events such as neuronal differentiation, axonal outgrowth, and the reestablishment of synaptic connections. Therefore, a diverse ECM microenvironment is required to orchestrate cell growth. Moreover, the tissue-specific effect of ECM can promote more site-appropriate phenotypic cell differentiation than the effect of ECM scaffolds derived from nonhomologous tissue sources [18, 19], and intact brain-VBM is believed to contain bioactive components and a microstructure that are uniquely able to induce constructive brain remodeling.

In the process of neural regeneration after brain injury, a suitable physical framework of biomaterials is required to provide a physical matrix for cell growth, axonal regeneration, and neurite outgrowth and maintain a homeostatic microenvironment by allowing diffusion of nutrients and metabolites and other soluble factors [20]. Our results illustrate that the brain-VBM has a randomly orientated, reticular structure with a high porosity (over 95%) that provides a high surface area for cell attachment and nutrient and oxygen delivery. The pore size ranges from 10 to 100 μm, fulfilling the requirements for tissue regeneration. Small pores typically provide physical protection by restricting inflammatory reaction exacerbation, thereby markedly enhancing the viability of immigrated neural cells, while large pores facilitate the infiltration of neural stem cells and neovascularization and the mass transportation of nutrients and oxygen. More importantly, for filament-like biomaterials, some researchers have reported that the distance between fibers influences the neurites extending from cells, and larger distances (> 15 μm) cause neurite alignment and migration along with the fibers, whereas smaller distances (< 15 μm) induce neurites to grow transversally [21]. The space between fibers in brain-VBM is almost always distributed in the range 30–50 μm, revealing a strong ability to create an optimal niche for the establishment of neural networks and thus restore the loss function of the injured brain. Moreover, the thicker fibers in brain-VBM facilitate cell attachment by providing more surface area.

Brain-VBM is an important part of brain-ECM. Although the total amount of protein in the processed brain-VBM was less than 10% of that in the original tissue, the concentrations of ECM-related proteins (such as collagen, LN, and FN) were 100–200 times higher in the processed brain-VBM than in the original tissue (Fig. 3), indicating that most of the ECM proteins are retained during processing. The ECM of brain tissue consists of two main sets of components: the components of the VBM, including collagen, LN isoforms, nidogens, and heparan sulfate proteoglycans, and the components of the brain parenchyma, including CSPGs and other glycoproteins. The VBM consists of structural proteins, including collagen type IV and LN, which maintain an intact three-dimensional structure, and matrix active proteins, including FN, nidogens, glycoproteins, and proteoglycans, which can serve as intercellular messengers. These two types of proteins [22, 23] play synergistic roles in the process of brain regeneration: (1) enhancing cell adhesion, migration, and proliferation; (2) strongly inducing axonal extension and synaptic plasticity (LN); and (3) acting as scaffolds supporting the migration of NPCs into the lesion site. Indeed, brain-VBM can act as a reservoir of active factors that can release or retain biological factors, such as cell adhesion molecules, under spatial and temporal control, providing more adhesion sites for cell (endogenous or exogenous) colonization and facilitating cell growth into the scaffolds [24]. Due to the high binding affinity, brain-VBM can carry more cells than other artificial biomaterials, representing an ideal carrier for cell transplantation.

New approaches to generate decellularized materials with minimal damage to brain-VBM in terms of structure and composition must be explored urgently. After many attempts, surprisingly, we eventually discovered an effective protocol in which weakly alkaline double-distilled water (pH 8.0–8.5) was the main reagent. As one of most common solutions used in the laboratory, sodium hydroxide (pH 8.0–8.5) showed a remarkable result when used for the extraction of brain vascular matrix. When combined with gentle agitation (orbital shaker, 60–80 rpm), this method prepared a satisfactory intact whole-brain VBM within 12 days (Fig. 1). The preparation process can be shortened to 5–8 h if the brain tissue is cut into pieces (e.g., 5 cm × 5 cm × 5 cm). Compared to conventional preparation techniques [25], which are extremely cumbersome processes consisting of filtration by continuous centrifugation and agitation with glass beads, our new preparation scheme is notable for its high efficiency and ease of execution. Our method uses only weakly alkaline double-distilled water without any other chemical agents, representing a physiologically benign processing condition that supports the biocompatibility of the resulting brain-VBM-based scaffolds. Conventional VBM preparation techniques typically require tissue homogenization with a tissue grinder [12, 26], which inevitably destroys the intact structure of the VBM and yields only a small amount of VBM fragments. As a result, the bioactivity of the VBM is largely lost, which diminishes the function of the VBM scaffold. In contrast, our procedure preserves the structure, chemical composition, and bioactivities of the VBM to a large extent.

The role of brain-VBM in promoting neural tissue regeneration after TBI is complex. In the early stages after VBM grafting in the rat TBI model, neural cells and endothelial cells continue to migrate and extend to the injured area with the help of brain-VBM. Repopulation of neuronal cells is the key to nerve regeneration. In the present study, after injury, NPCs (such as RGPs in this study) and DCX^+^ neurons continuously migrated from other regions to the injured region under the influence of the structure and composition of the brain-VBM (7 days after injury, neurons in the dentate gyrus were activated and DCX^+^ neurons had migrated to the lesion sites). These neurons eventually matured and functioned. New axons in the lesion area establish connections or synaptic connections with axons already present in the host tissue to reconstruct the damaged neural network in the lesion area. Due to its own intact and continuous structure, brain-VBM provides a physical substrate for neuronal cells to migrate to the lesion area and helps cells and axons cross the lesion boundary. Our results (Fig. 7) demonstrated that Nestin-positive cell populations were present in the brain-VBM 20 days after TBI, which may be due to the specific physical properties of the intact VBM (e.g., stiffness, elasticity, topography) and ECM components (integrins, FN, collagen IV, LN, and sequestered growth factors) that create a suitable niche for NPCs.

A previous study [25] demonstrated that brain-ECM components induced abundant neovascularization in chick embryonic chorioallantoic membranes and had the same effect as VEGF. Although that study did not examine the concentrations of relevant active factors, we speculate that the residual active VEGF component in the graft may be an important vascular induction factor. Our in vitro data confirmed that brain-VBM contains many vasoactive components, such as VEGF, that remained during the preparation process, indicating that brain-VBM is likely to have a strong ability to induce angiogenesis. The results of this study also revealed that brain-VBM rapidly induces neovascularization (Fig. 9A) and establishes blood supply at the edge of the lesion as early as 10 days after grafting, which helps create a permissive microenvironment to induce the migration of neuronal cells and newly generated axons to the center of the lesion site while providing nutrition for the survival and maturation of newly generated neurons, which may explain why a considerable number of new blood vessels are closely associated with neurons (Fig. 8E). However, several previous studies [27–29] have reported that under normal conditions, nerves may actually guide the growth of blood vessels during normal development in vivo. Thus, whether neuronal axons promote the recruitment of endothelial cells or whether endothelial cells promote the directed outgrowth of neurons at the site of injury is unclear; whether the effects are direct or indirect is also unknown. Nevertheless, angiogenesis provides rich nutritional support for new neurons and axons to extend through the lesion region and plays an important role in improving the local microenvironment.

Tissue engineering has contributed extensively to the field of neural repair and regeneration. Synthetic scaffolds [16, 30] were overly simple compared to brain-ECM networks due to the spatial and component uniformity produced by mechanical technology. Although existing artificial biomaterials have been designed to structurally and constituently mimic the natural ECM of brain tissue, these synthetic materials often lack bioactivity and the capacity for an integrative host tissue response, resulting in fibrous encapsulation [31]. In fact, we believe that the brain-ECM in the present study can recapitulate the features (including the structure and composition) of the real, natural ECM from neural tissue and can therefore be used as a biological material for many aspects of neuroscience research.

Notably, genipin-crosslinked brain-VBM possesses good memory properties. Even if the volume is compressed to 1/20 of the initial volume, the deformation can still be recovered (> 97%) in a short time (< 5 s). Most 3D materials with a shape memory function are artificially synthesized [32, 33]. In the study, we report the first natural material with shape memory properties. We envision that as an injectable 3D scaffold or hydrogel, VBM may have a wide range of applications in minimally invasive clinical procedures. For deep brain lesions, brain-VBM can be delivered to the defect site through guided stereotactic injection (see video 2). Rehydration followed by expansion of the material may markedly facilitate minimally invasive clinical delivery.

As a novel functional material, brain-VBM presents a tissue-specific complex structural and functional ECM composition that still faces many limitations prior to clinical use, including the potential risks to trigger immune responses in vivo, poor mechanical properties, and uncontrollable degradation. Ongoing research is seeking to address all of these issues and refine brain-VBM for cell delivery and transplantation in clinical practice*.*

## Conclusion

Brain-VBM is an important component of the brain ECM and is considered a novel, potentially multifunctional 3D biomaterial because it preserves the 3D structure of the brain microvasculature. However, no technique for isolating structurally and compositionally intact brain-VBM is available. This study introduces a novel method for extracting structurally intact brain-VBM with a simple, safe, and effective procedure. This 3D porous network scaffold provides a physical matrix and active factors for cell survival that will further promote cell proliferation along with axonal extension and regeneration. In the in vivo experiments, brain-VBM facilitated neural regeneration after TBI, and its promotion of angiogenesis may be a very important reason. As a novel 3D scaffold with a naturally active structure for CNS repair, brain-VBM provides new materials and options for neural tissue engineering. However, research on brain-VBM is still preliminary, and the technology faces many obstacles to its application, such as low stability, limited control over mechanical properties, and rapid biodegradation. Many interesting questions remain with respect to brain-VBM as a scaffold biomaterial to support tissue regeneration and assist in cell therapy and minimally invasive surgery.

## Experimental section

### Animals

A total of 60 adult Sprague-Dawley rats (body weight, 180–200 g; age, 8 weeks) were used in this study. Twenty rats provided the raw materials for brain-VBM samples, and the other 40 rats were used to establish the traumatic brain injury model. All experimental animals (provided by the Experimental Animal Center of the School of Medicine, Sun Yat-sen University) were housed in temperature- and humidity-controlled animal quarters under a 12-h light/dark cycle. All protocols were approved by the Experimental Animal Administration and Ethics Committee of Sun Yat-sen University, Guangdong, China.

### Brain-VBM preparation

The rats were euthanized under deep anesthesia, and the entire brain tissue (or other parts of the CNS) was isolated in the shortest possible time. The surface of the brain tissue was kept moist by phosphate-buffered saline (PBS) (0.01 M; pH 7.2–7.4) during the process. Under special circumstances, the tissue could be divided into tissue blocks of varying sizes (such as 5 mm × 5 mm × 5 mm) according to the anatomical characteristics before the following procedures.

#### Extraction treatment

The specimens were washed with deionized water three times, 5 min each time, and transferred to a 10-cm glass Petri dish. The entire tissue specimens were soaked in weakly alkaline water (prepared with double-distilled (dd) water + sodium hydroxide, pH = 8–8.5), and the liquid in the Petri dish was replaced with fresh weakly alkaline water (pH = 8–8.5) every 15–30 min to keep liquid clear and transparent. The processing time was determined by the size and source of the specimen. When the specimens were completely transparent, they were washed with PBS (0.01 M; pH 7.2–7.4) three times for 10 min each time. The specimens were then treated with 1000 Kunitz units of DNase type I (Amresco, Shanghai, China) and 25 mg/mL ribonuclease (Amresco, Shanghai, China) for 1 h at 37 °C, followed by three washes with PBS (0.01 M; pH 7.2–7.4) for 10 min each. The entire process was carried out under continuous shaking conditions (60–80 rpm; room temperature).

#### Crosslinking and sterilization

To increase the mechanical strength, the alkaline-treated specimens were transferred into pre-prepared genipin solution (0.5%, Wako, Japan) in the dark at room temperature for 72 h. The crosslinked specimens were washed with PBS (0.01 M; pH 7.2–7.4) on a shaker (60 rpm) three times for 3 min. The washed specimens were sterilized in 1‰ peroxyacetic acid (PAA, St. Louis, MO) for 6 h and were then washed with sterile PBS (0.01 M; pH 7.2–7.4) three times for 3 min. The final samples were stored in sterile PBS solution (0.01 M; pH = 8) at 4 °C. Some specimens were freeze–dried with the FreeZone Freeze Dry System (Labconco, US) for later use.

### Characterization of brain-VBM

#### Structure

##### Light microscopy and H&E staining

The microstructure of the final product processed in section 2.2 in a fully extended state in PBS solution was examined under an ordinary light microscope. Then, the specimen was embedded in Tissue-Tek® OCT Compound (Sakura Finetek, USA), and cryostat sections with a thickness of 5–10 μm were prepared with a cryostat (CryoStar™ NX70, US). The slices were stained with H&E and imaged with the Eclipse 80i Digital Imaging Head Fluorescence Microscope (Nikon, Japan). The results were analyzed in Image-Pro Plus 6.0 software (Media Cybernetics, Inc.).

##### Transmission electron microscopy (TEM) and toluidine blue

The specimens to be examined were fixed with 3% glutaraldehyde for 6 h at 4 °C, washed with 0.01 M PBS (pH 7.2–7.4) six times for 3 min, and postfixed in 4% osmic acid for 2 h at 4 °C, followed by acetone gradient dehydration. The dehydrated specimens were embedded in Poly/Bed 812 resin (Ted Pella, CA, USA) and polymerized at 60 °C for 48 h. The following sections were prepared with an ultrathin microtome (Leica EM UC7-Leica): (1) semithin sections (0.5–1 μm) for toluidine blue staining, which were stained with 0.1% toluidine blue O and imaged under the Nikon Eclipse 80i Digital Imaging Head Fluorescence Microscope at the magnification of 800×; (2) ultrathin sections (60–80 nm) for TEM, which were mounted onto a 200-mesh formvar-coated copper mesh, stained with uranyl acetate and lead citrate, and examined by TEM (FEI TECNAI G^2^ SPIRIT).

##### SEM

For the SEM analysis, the specimens were fixed with glutaraldehyde and 5% osmium tetroxide for 2 h respectively, dehydrated with 70, 80, 90, and 100% ethanol, soaked in isopentyl acetate for 15 min to replace the ethanol in the specimen, and dried with the critical-point drying method (Hitachi, Tokyo, Japan) for 4 h. The dried specimen was coated with gold, and the microstructure of the scaffolds was examined by the Quanta 200 SEM (FEI, USA). The images of toluidine blue–stained sections and the TEM and SEM images were imported into Image-Pro Plus 6.0 (Media Cybernetics, Inc.) to measure the diameters of pores and filaments within the scaffold.

##### Porosity

The porosity of the tissue was examined by the ethanol substitution method. The freeze–dried specimens to be examined were randomly divided into three groups (*N* = 10), and all the specimens in each group were placed in a volume (V1) of ethanol. When the specimens were completely soaked, the volume of ethanol was recorded (V2). The volume of the specimen itself was thus V2 − V1. The specimen was immediately removed, and the remaining volume of ethanol was recorded (V3). V1 − V3 was the volume of ethanol that could be absorbed by the specimens to be examined (V_ethanol_). The total volume of the specimens (V total = occupied ethanol in the specimens and the intrinsic volumes of the specimens) = (V2 − V1) + (V1 − V3) = V2 − V3. The porosity of the specimens was calculated by V_ethanol_/V_total_.

##### Determination of shape recovery rate and recovery time (RT)

The crosslinked specimens to be examined (size: 1 cm × 1 cm × 5 mm) were completely soaked in PBS (0.01 M; pH = 8.5). After aspiration with minicaps (30 μL; inner diameter: 1.02 mm; HIRSCHMANN; Germany), the specimens were injected into PBS through the 1.02-mm orifice. The entire injection process was recorded by a Canon EOS 80D high-speed imaging system. The specimen dimensions before and after aspiration and injection were measured and calculated using a digital micrometer. Shape recovery rate = V_before aspiration_/V_after injection_. The time required for structural recovery was calculated using the frame rate (frames per second) of the recorded videos.

##### Measurement of mechanical properties

The mechanical properties of brain-VBM were assessed by uniaxial tension tests with Zwick (Z005, German) testing equipment. The specimens were placed between the pneumatic grips and subjected to a load cell of 10 N under ambient conditions. At least three groups of specimens were measured (*n* = 5), and the corresponding data (including elastic modulus, ultimate tensile strength, and ultimate elongation of specimens) were recorded as the mean values of these measurements.

#### Component analysis

##### DNA electrophoresis

Qualitative assessment of residual DNA was conducted by digestion of the brain-VBM in 0.1 mg/mL proteinase K (for 48 h at 37 °C). The protein was removed through phenol–chloroform extraction. The supernatant after centrifugation was mixed with 3 M sodium acetate and 100% ethanol. DNA was acquired as the pellet following centrifugation and then rinsed with 70% ethanol, centrifuged, and dried. Double-stranded DNA was quantified using the Quant-iT™ PicoGreen™ dsDNA Assay (ThermoFisher; No: P11496). The base pair length of residual DNA in the transparent tissue was determined by gel electrophoresis: Tissue DNA Kits (Omega Bio-Tek; Guangzhou; China) were used to extract DNA from 30-mg tissue specimens. Agarose gels (1%) were loaded with DNA and electrophoresed for 1 h at 60–100 V; 4 μg/mL Gel Green (Biotium, Fremont, California, USA) was used for post-electrophoresis gel staining for 30 min, and the gels were visualized in a 254-nm UV transilluminator.

##### Sodium dodecyl sulfate–polyacrylamide gel electrophoresis (SDS-PAGE)

The specimens were centrifuged at 3000 rpm for 3 min, and the supernatants were discarded. The pellets were homogenized with 300 μL SDT lysis buffer, incubated in boiling water for 5 min, sonicated (80 W, working 10 s, interval 15 s, for 10 cycles), incubated in boiling water for 15 min, and centrifuged at 14,000 *g* for 40 min. Proteins were quantified by the bicinchoninic acid assay, aliquoted, and stored at − 80 °C. Ten micrograms of protein specimen was mixed with the loading buffer, incubated in boiling water for 5 min, separated by 12.5% SDS-PAGE (constant current 14 mA, 90 min), and stained with Coomassie brilliant blue.

##### Fluorescence immunostaining

After being embedded in Tissue-Tek® OCT Compound (Sakura Finetek USA), specimens were sectioned (5–10 μm) using the cryostat (CryoStar™ NX70, US). The slides were washed with PBS three times for 3 min, blocked in normal goat serum for 1 h at 37 °C, incubated in the primary antibody (rabbit anti-laminin, 1:1000, Sigma-Aldrich, St. Louis, MO; rabbit anti-Collagen 1:500, Abcam, Cambridge, MA, US; anti-fibronectin, 1:400, Abcam, Cambridge, MA, US; mouse anti-chondroitin sulfate proteoglycan antibody, 1:1000, Catalogue Number: MAB1581, Chemicon) at 4 °C overnight, washed with PBS three times for 3 min to remove the residual primary antibody, and then incubated in the corresponding secondary antibody (Jackson ImmunoResearch Inc.; West Grove, PA, USA), including Alex Fluor 488-conjugated and Alex Fluor 594-conjugated secondary antibodies, at 37 °C for 1 h. After washing with PBS three times for 3 min, the slides were counterstained with 25 μg/mL 4′,6-diamidino-2-phenylindole (DAPI) (Sigma-Aldrich) and imaged with the Nikon Eclipse 80i Digital Imaging Head Fluorescence Microscope.

##### Confocal 3D structure reconstruction

Specimens were incubated in a primary antibody solution for 24 h at 4 °C, washed with PBS three times for 10 min on a shaker, and incubated in the Alex Fluor 488-conjugated secondary antibody (Jackson ImmunoResearch Inc.; West Grove, PA, USA). Specimens were then examined and imaged under a laser scanning microscope (LSM880 with Fast Airyscan, ZEISS, Germany). The data were analyzed using ZEN lite software to observe and rebuild the 3D view.

##### Examination of protein expression profiles by enzyme-linked immunosorbent assay (ELISA)

Specimens were isolated, cut into small fragments, homogenized, and incubated in cold lysis buffer (20 mM HEPES, 0.5 mM ethylene glycol bis (beta-aminoethyl ether)-N,N,N′,N′-tetraacetic acid, 1 mM dithiothreitol, and 0.32 M sucrose; pH 7.4) containing phenylmethanesulfonyl fluoride (protease inhibitors; LiankeBio; Hangzhou) for 30 min at 4 °C. Then they were centrifuged at 3000 rpm for 5 min at 4 °C. The supernatant was collected for examination. According to the manufacturers’ protocols, relevant active factors were quantified with the Soluble Collagen Assay Kit (Abcam, Cambridge, MA, US; ab241015), Rat Integrin-β1 ELISA Kit (Cusabio Biotech, Wuhan, China, code: CSB-E14205r), Rat E-Cadherin ELISA Kit (Abcam, Cambridge, MA, US; ab202413), Rat VEGF Quantikine ELISA Kit (R&D Systems Inc., Minneapolis, MN, USA; Catalog #:MFB00), Rat FGF2 Quantikine ELISA Kit (R&D Systems Inc., Minneapolis, MN, USA; Catalog #:MFB00), and Rat beta NGF ELISA Kit (Abcam, Cambridge, MA, US; ab193736). The signal was recorded by a microplate reader (Bio-Rad; USA), and the optical density measured at 450 nm (OD_450_) was used to calculate the concentration of the protein of interest in the specimen.

#### Proteomics

##### Protein extraction and peptide digestion specimens

Proteins were extracted by the SDT (4% (w/v) SDS, 100 mM Tris/HCl (pH 7.6), 0.1 M dithiothreitol) lysis method and quantified by the bicinchoninic acid assay. The filter-aided proteome preparation method was used for trypsin digestion, and the peptide was desalted by a C18 cartridge. The peptide was lyophilized and reconstituted with 40 mL 0.1% formic acid and quantified (OD_280_).

##### LC-MS/MS data collection

Each specimen was separated by a high-performance liquid chromatograph, Easy-nLC, with a nanoliter flow rate. Buffer A was a 0.1% formic acid aqueous solution, and buffer B was a 0.1% formate–acetonitrile aqueous solution (with 84% acetonitrile). The chromatographic columns were balanced with 95% buffer A. Specimens were loaded to the loading columns by an automatic sampler (Thermo Scientific Acclaim PepMap100, 100 μm × 2 cm, nanoViper C18) and separated through an analytical column (Thermo Scientific EASY column, 10 cm, ID 75 μm, 3 μm, C18-A2) at the flow rate of 300 nL/min. After chromatographic separation, the specimens were analyzed using the Q-Exactive mass spectrometer by detecting the positive ions, the scanning range of the precursor ions being 300–1800 m/z, the resolution of the primary mass spectrometry being 70,000 at 200 m/z, the automatic gain control target being 1e^6^, the maximum ion trap being 50 ms, and the dynamic exclusion being 60.0 s. The mass-to-charge ratios of polypeptides and polypeptide fragments were collected according to the following method: 20 fragment maps were collected (MS2 scan) after each full scan with the MS2 activation type of higher-energy C-trap dissociation, the isolation window of 2 m/z, the tandem mass spectrometry resolution of 17,500 at 200 m/z, the normalized collision energy of 30 eV, and the underfill of 0.1%. The raw data were input into MaxQuant (version 1.5.3.17) for protein identification and quantitative analysis. Label-free quantitation intensity was used to compare the relative protein expression between groups.

### Cell scaffold composition and biocompatibility analysis

#### Cells

BMSCs were isolated according to a previous study from hematopoietic cells from the left femur of each animal (which continued to be used in subsequent in vivo experiments). The rat primary cortical neurons were purchased from XinPeng Biotechnology Co., Ltd. (Guangzhou, China).

#### Cell seeding

Crosslinked and sterilized specimens were placed in 96-well plates (the size of the brain-VBM was 5 × 5 × 2 mm in each well) and incubated overnight with α-minimal essential medium (MEM) at 37 °C in 5% CO_2_. A total of 100 μL (1 × 10^7^ cells/mL) of the cell suspension was gradually dropped onto the brain-VBM in each well, and the plate was incubated for 3 h at 37° in 5% CO_2_. Each well was supplemented with 1 mL culture medium, and the plate was cultured for another 24 h. Then, unattached cells were removed by gently washing with sterile PBS three times for 30 s. Brain-VBM samples with attached BMSCs were transferred to 96-well plates, cultured in α-MEM containing 10% fetal bovine serum for 4 days, and subjected to the following tests.

#### Biocompatibility analysis

##### Cell distribution on brain-VBM

To examine the distribution and morphology of cells on brain-VBM, after 4 days of incubation, MSCs seeded on brain-VBM were washed with PBS, and then fixed for 2 h in 10% neutral buffered formalin solution (pH 7.4). After cryosectioning at 10 μm, the slices were washed with Hanks’ Balanced Salt Solution (HBSS) and stained with 25 μg/mL DAPI (Sigma-Aldrich) to determine the distribution of cells on the brain-VBM scaffolds. Some other constructs were fixed with glutaraldehyde (3% (v/v)) for SEM (refer to the relevant section for detailed methods).

##### The impact of brain-VBM on cell proliferation and axon growth

Lyophilized and comminuted brain-VBM (1 g/mL) was placed in 0.01 N hydrochloric acid solution containing 1 mg/mL pepsin (Sigma). The pepsin was allowed to digest the brain-VBM for 48 h under constant stirring (60 rpm) at 4 °C. After deactivation of pepsin with PBS (pH = 7.5), brain-VBM was diluted to the desired concentrations with 1× PBS for subsequent experiments. Mitogenic effects (MSCs) and the effect on neurite outgrowth (cortical neurons) of the components of brain-VBM were assayed.

To examine the potential cytotoxic effects of the components of brain-VBM, BMSCs were seeded in 48-well plates (1 × 10^4^ cells/well). After cell attachment, a range of concentrations of brain-VBM was added to the medium (final concentration 2.5 mg/mL, 10 mg/mL, 25 mg/mL, or 100 mg/mL), and cells were cocultured with brain-VBM for an additional 48 h. Cultures in each well were treated with 200 μL (v/v: 10:1) Dulbecco’s modified Eagle’s medium-F12 medium and methyl thiazolyl tetrazolium (MTT) solution (5 mg/mL in PBS), and incubated for 4 h at 37 °C. The medium was then replaced with 150 μL dimethyl sulfoxide (Sigma-Aldrich; D2650), and the plate was gently shaken at room temperature for 12 min. The OD_490_ of each well was measured in a microtiter plate reader.

Cortical neurons were isolated from rats at postnatal day 1 and plated into cell culture flasks precoated with poly-l-lysine (500 μg/mL Sigma-Aldrich). Cells were allowed to attach at 37° under 5% CO_2_ and to grow in medium (Gibco, USA) supplemented with 2% B27 and 0.5% l-glutamine. Two weeks later, a range of concentrations of brain-VBM were added to the medium, and the effects of brain-VBM on neuronal axon growth were assessed by immunofluorescence staining for NF-200.

The mRNA levels of NGF-β and BDGF in cortical neurons at different coculture times (12 h, 24 h, 36 h and 48 h) were analyzed by reverse transcription polymerase chain reaction (RT-PCR). β-Actin mRNA was used as an internal control. Total RNA from each well was isolated by TRIzol (Takara Bio, Japan), and then a two-step PCR amplification was performed according to the manufacturer’s instructions. Amplification products were separated by 2.0% agarose gel electrophoresis and visualized by GelGreen (4 μg/mL, Biotium, Fremont, California, USA). The relative intensity of the bands was determined using a 254-nm UV transilluminator (Labworks 4.6) and analysis software (UVP, Inc.). The experiments were repeated three times.

The primer sequences for NGF, BDGF, and β-actin are listed in Table 1.

**Table 1:** The primer sequences for NGF, BDGF, and β-actin

Primer	Forward primer	Reverse primer
NGF-β	5′-GGCCACTCTGAGGTGCATAG-3’	5′-CATGGGCCTGGAAGTCTAAA-3’
BDNF	5′-AAACCATAAGGACGCGGACT-3’	5′-GATTGGGTAGTTCGGCATTG-3’
β-actin	5′-TCTACGAGGGCTATGCTCTCC-3’	5′-GGATGCCACAGGATTCCATAC-3’

##### Live/dead cell assay

The viability of BMSCs seeded on brain-VBM was assessed with the LIVE/DEAD™ Cell Imaging Kit (Invitrogen, Thermo Fisher Scientific, Cat. No: R37601). After 4 days of incubation, cells were incubated in the live (green)/dead (red) reagent for 15 min (at 20–25 °C) and examined under a laser scanning microscope (LSM880 with Fast Airyscan, ZEISS, Germany). ZEN lite software was used to manage and measure microscope images (Orthogonal view). The results were analyzed in Image-Pro Plus 6.0 software (Media Cybernetics, Inc.).

### In vivo brain injury model and surgical procedure

Sprague-Dawley rats (220–250 g), half male and half female, were used for this study. After anesthesia with an intraperitoneal injection of pentobarbital (6.5 mg/100 g body weight), each experimental rat was placed in the prone position in a stereotaxic apparatus and underwent the following surgical procedure in an aseptic environment. Sham group (*N* = 10): After craniotomy, the bone flap was separated and restored. The integrity of the dura mater was not undermined during the process. Experimental group (*N* = 15): After craniotomy, the brain tissue of the left frontal lobe was exposed. The dura mater was carefully cut by microscissors, and a cortical lesion cavity (length 5 mm × width 5 mm × depth 2 mm) was created from 1.0 mm anterior to 4.0 mm posterior to the bregma and 3 mm lateral to the midline. A block of brain-VBM was placed into the lesion cavity after cortex removal, with the volume of the implanted specimen filled the entire cavity. To prevent later tissue adhesion, the defective dura mater was repaired with a latex biomembrane before restoring the bone flap. For the blank control group (*N* = 15), the lesion cavity was filled with PBS.

During the surgical procedure, bleeding was stopped by local compression with sterile cotton soaked with epinephrine and saline (V/V; 1:200,000 to 500,000). The skin was sutured using 4–0 silk (Ethicon) with a reverse cutting needle. The above procedure was carried out under an sXP-10 microscope (Leica, USA) at 25× magnification. After surgery, the rats were returned to their home cages (under standard conditions) and fasted on the first day. They had no dietary restrictions afterward. The rats were treated with an antibiotic (ceftriaxone sodium; 5 mg/kg; Roche) by intraperitoneal injection for three consecutive days to prevent bacterial infection. The recovery of the animals was closely monitored.

### Assessment of neurological functional recovery

Researchers who were blinded to the experimental groups performed the modified neurological severity score (mNSS) assessment before the surgery and every other week from the first week after the surgery to determine the recovery of neurological functions. The mNSS includes motor, sensory, balance, and reflex tests. A higher score reflects more severe injury (the score ranges from 0 to 18 points, 0 points being normal and 18 points being the maximal deficit score).

### Morphological observation of tissue regeneration

Brain tissue was harvested at the indicated time points after surgery according to the following procedure. The rats were injected with a lethal dose of anesthetic and transcardially perfused with 200 mL ice-cold paraformaldehyde (4%). The brains were isolated and stored in 30% sucrose solution overnight for cryoprotection. Brains were then sectioned with a microtome (RM2145, Leica) for experiments.

#### H&E staining

Serial sections (20 μm) through the cerebral cortex were stained with H&E to measure the areas of the brain cavity after TBI.

#### TEM

After some specimens were taken out, they were rapidly fixed with osmium tetroxide and glutaraldehyde. Semithin and ultrathin sections were double-stained with 3% uranyl acetate–lead citrate and examined through TEM to assess the ultrastructural changes in the regenerated nerve tissues and vascularization.

#### Immunohistochemical analysis

The sections (10-μm) were incubated in blocking solution with primary antibodies: anti-nestin (1:400; MAB353, Millipore, Billerica, MA, USA) as a marker of neural stem cells (NSCs) and neural progenitor cells (NPCs), anti-glial fibrillary acidic protein (GFAP) (1:500; No. 3670, Cell Signaling Technology, MA, United States) as a marker of actively dividing astrocytes, anti-neurofilament 200 (NF-200; 1:2000; Sigma) to label neuronal axons, anti-NeuN (1:800; MAB377; Millipore) as a marker of mature neurons, anti-doublecortin (DCX; 1:1000, 4604S, Cell Signaling Technology), a microtubule-associated protein primarily expressed by migratory immature neurons and neuronal neural progenitors, is widely used as a marker of adult neurogenesis, anti-CD68 (Cell Signaling Technology) as a marker of macrophages. Alexa Fluor 594-conjugated or Alex Fluor 488-conjugated secondary antibodies were used for detection. Nuclei were counterstained with 25 μg/mL DAPI (Sigma-Aldrich). Images were taken by a fluorescence microscope (Eclipse 80i, Nikon) equipped with a high-resolution color digital camera (Digital Sight US-U2, Nikon) The results were analyzed using Image-Pro Plus software (Media Cybernetics, Inc.).

### Statistical analysis

The data are presented as the mean ± SD. Data from two groups were assessed by the two-tailed unpaired t-test for any significant differences. Comparisons between multiple groups were assessed by one-way analysis of variance and the Bonferroni method of multiple comparisons. *P* < 0.05 was considered to indicate statistical significance. GraphPad Prism 8.0 (GraphPad Software Inc., San Diego, CA, USA) was used for all statistical analyses.

## Supplementary Information

**Additional file 1.**

**Additional file 2.**

**Additional file 3: Table1.** Advantages and disadvantages of biomaterials for neural tissue regeneration.

**Additional file 4: Figure S1.** A After treatment, the entire brain became completely transparent on day 12. B After extraction, DAPI stain revealed the brain-ECM retained a small portion of DNA remnant without D/RNase treatment. C 2 hours after coculture, phase contrast Microscope confirmed brain-VBM have great cell adhesive properties. D Immature neurons(migrant neurons) were found in the dentate gyrus zone (SGZ) with a migratory stream to the lesion site. E renascent axons (yellow arrows shown growth cones ) tried to pass through and enter the lesion area. F At the early stage(10day after operation), astrocytes were confined around the lesion site, only small amount of them entered into the injury area. G After crosslinking, the brain-VBM exhibited a uniformly light blue color with genipin Scale bar:100μm.

**Additional file 5: Figure S2.** Confocal fluorescence imaging showed the survival rate of BMSCs at the different sites of brain-VBM 2 days after cell seeding.Red box shown central site and carmine box shown peripheral site. The number at the bottow of images show dimension labels of Z-stack.

**Additional file 6: Figure S3.** Bioinformatic analysis of the proteins in brain-VBM and normal brain tissue showed the following: The enriched GO terms (B) showed that the protein functional characteristics were all associated with ECM, cell binding, and cell adhesion. KEGG pathway enrichment(C) showed the 20 cellular signaling pathways with the most differentially expressed proteins. A shows the cluster analysis of differentially expressed proteins.

## Data Availability

Data sharing is not applicable to this article as no new data were created or analyzed in this study.

## Abbreviations

Brain-VBM: Brain vascular basement membrane
CNS: Central nervous system
ECM: Extracellular matrix
H&E: Hematoxylin-eosin
TEM: Transmission electron microscopy
SEM: Scanning electron microscopy
RT: Recovery time
BMSCs: Bone marrow-derived mesenchymal stem cells
