# Supplementary material for: A simple method to isolate structurally and chemically intact brain vascular basement membrane for neural regeneration following traumatic brain injury
Source: Biomater Res. 2023 Jan 12;27:2. doi: 10.1186/s40824-023-00341-6 (PMC9837976; doi:10.1186/s40824-023-00341-6)
Supplement: Supplementary file 3 — Additional file 3: Table1. Advantages and disadvantages of biomaterials for neural tissue regeneration. [file 40824_2023_341_MOESM3_ESM.docx]

**A Simple Method to Isolate Structurally and Chemically Intact Brain Vascular Basement Membrane for Neural Regeneration Following Traumatic Brain Injury**

Wanqing Ji, Zhuopeng Ye, Hengxin Tang, Bo Xue, Zhenming Tian, Jiaming Wen, Yueyang Ba, Zhuopeng Chen, Ning Zhang*, Xuejun Wen*, Bo Hou*

Table1:Advantages and disadvantages of biomaterials for neural tissue regeneration.

| Biomaterial | Advantages | Disadvantages |
| --- | --- | --- |
| **Natural Material:**  Chitosan  Fibrin  Gelatin  Collagen  Hyaluronic acid  Alginate | - Biocompatible - Nontoxic - Biofunctional - Bioadhesive - Good cell recognition | - Rapid degradation rate - Difficult to maintain integrity - In vivo - Poor mechanical properties |
| **Synthetic Material:**  Polyethylene glycol (PEG)  Polylactic acid (PLA)  Poly (lactic-co-glycolic) acid (PLGA) | - Good mechanical strength - Can be processed in various ways - Able to seed cells/growth factors - Easily functionalized | - Slow degradation rate - Nonadherence of cells - Poor cellular response - Acidic byproducts |

**FigureS1 A**

After treatment, the entire brain became completely transparent on day 12

**FigureS1 B**

After extraction, DAPI stain revealed the brain-ECM retained a small portion of DNA remnant without D/RNase treatment

**FigureS1 C**

2 hours after coculture，phase contrast Microscope confirmed brain-VBM have great cell adhesive properties

**FigureS1 D**

Immature neurons(migrant neurons) were found in the dentate gyrus zone (SGZ) with a migratory stream to the lesion site

**FigureS1 E**

renascent axons (yellow arrows shown growth cones ) tried to pass through and enter the lesion area

**FigureS1 F**

At the early stage(10day after operation), astrocytes were confined around the lesion site, only small amount of them entered into the injury area.

**FigureS1 G**

After crosslinking，the brain-VBM exhibited a uniformly light blue color with genipin

Scale bar:100μm

**FigureS2:**

Confocal fluorescence imaging showed the survival rate of BMSCs at the different sites of brain-VBM 2 days after cell seeding.Red box shown central site and carmine box shown peripheral site. The number at the bottow of images show dimension labels of Z-stack

**FigureS3:**

Bioinformatic analysis of the proteins in brain-VBM and normal brain tissue showed the following: The enriched GO terms (**B**) showed that the protein functional characteristics were all associated with ECM, cell binding, and cell adhesion. KEGG pathway enrichment(**C**) showed the 20 cellular signaling pathways with the most differentially expressed proteins. **A** shows the cluster analysis of differentially expressed proteins.
