# Supplementary figures and images for: A simple method to isolate structurally and chemically intact brain vascular basement membrane for neural regeneration following traumatic brain injury
Source: Biomater Res. 2023 Jan 12;27:2. doi: 10.1186/s40824-023-00341-6 (PMC9837976; doi:10.1186/s40824-023-00341-6)

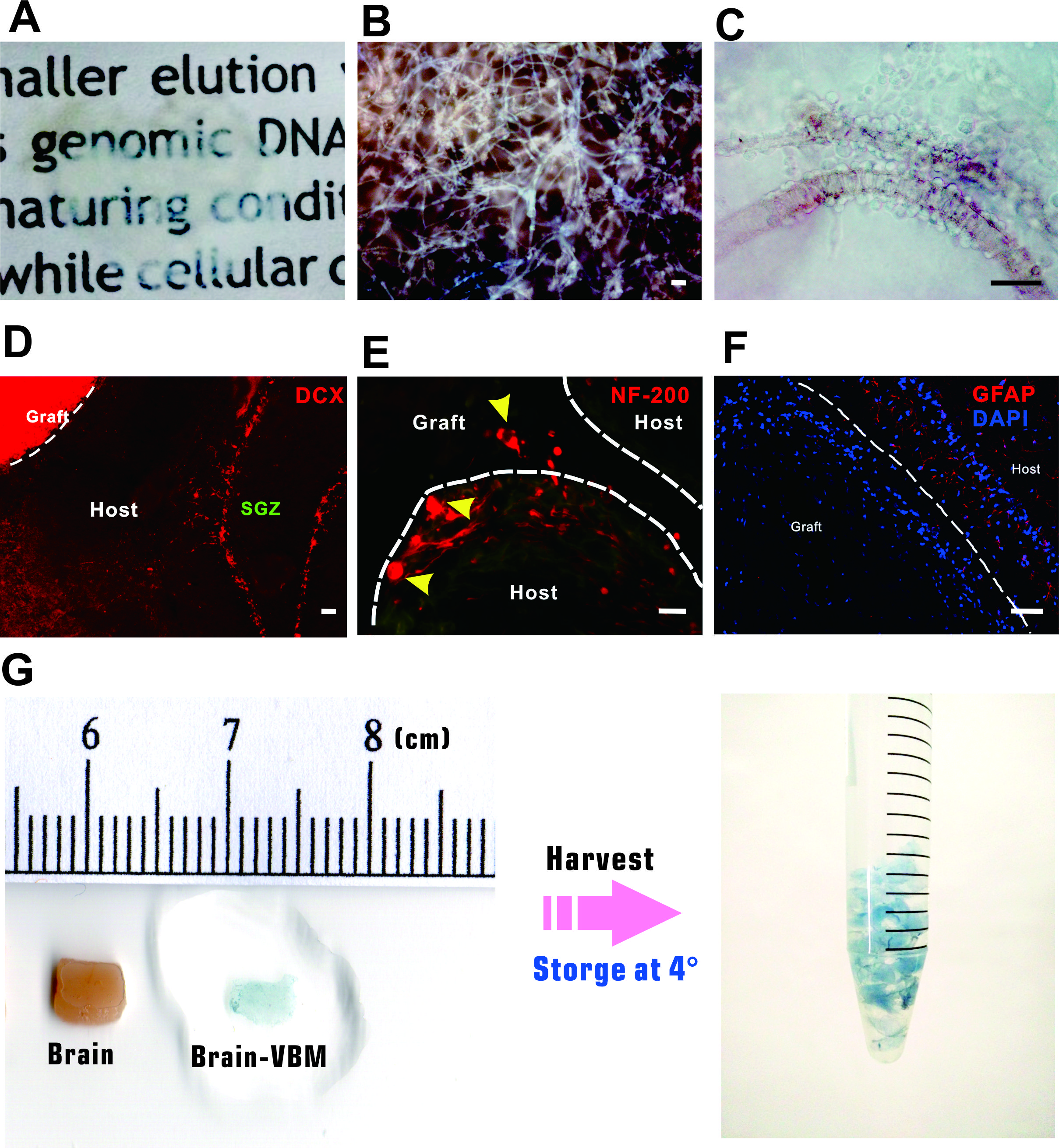

Supplement: Supplementary file 4 — Additional file 4: Figure S1. A After treatment, the entire brain became completely transparent on day 12. B After extraction, DAPI stain revealed the brain-ECM retained a small portion of DNA remnant without D/RNase treatment. C 2 hours after coculture, phase contrast Microscope confirmed brain-VBM have great cell adhesive properties. D Immature neurons(migrant neurons) were found in the dentate gyrus zone (SGZ) with a migratory stream to the lesion site. E renascent axons (yellow arrows shown growth cones ) tried to pass through and enter the lesion area. F At the early stage(10day after operation), astrocytes were confined around the lesion site, only small amount of them entered into the injury area. G After crosslinking, the brain-VBM exhibited a uniformly light blue color with genipin Scale bar:100μm. [file 40824_2023_341_MOESM4_ESM.jpg]

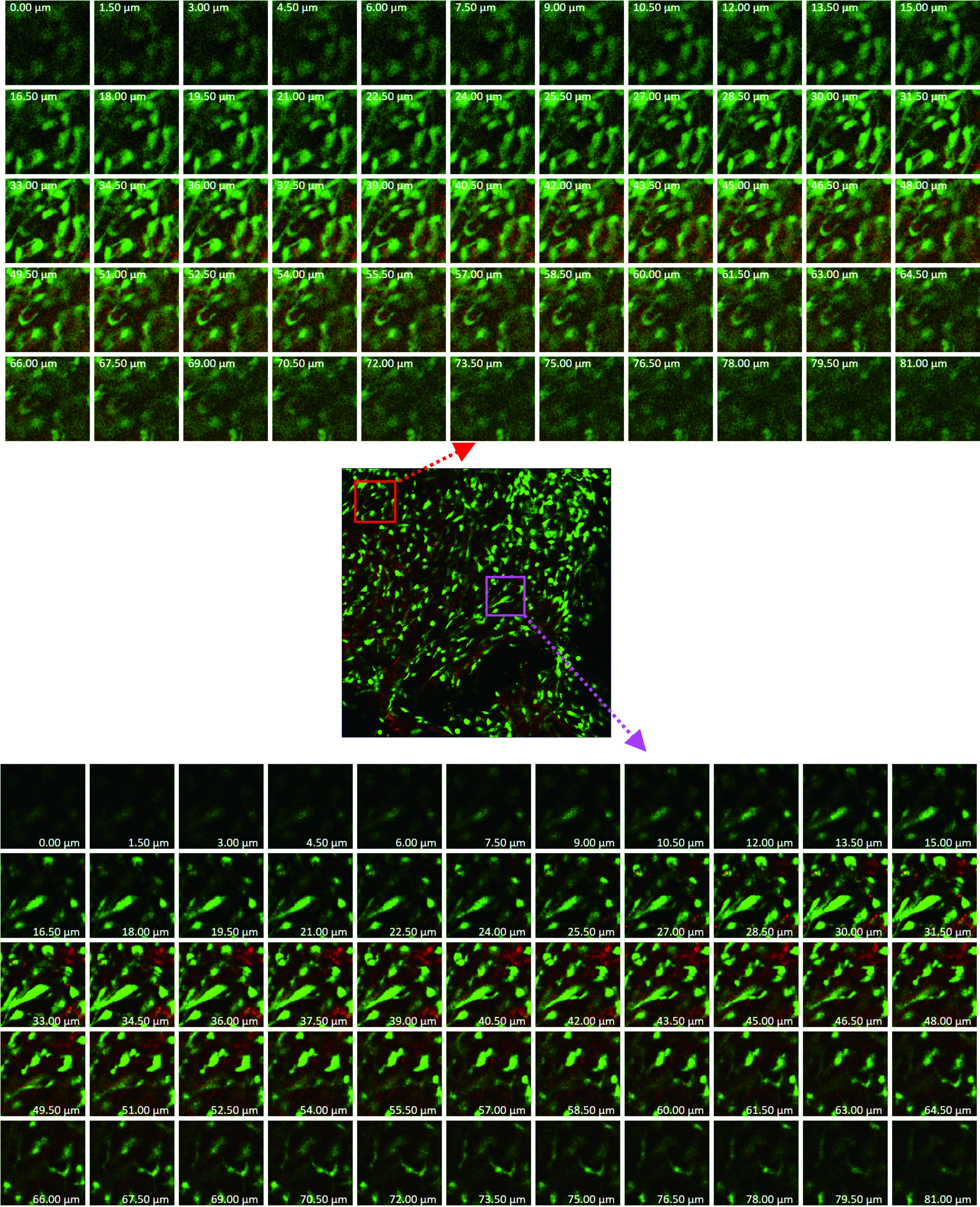

Supplement: Supplementary file 5 — Additional file 5: Figure S2. Confocal fluorescence imaging showed the survival rate of BMSCs at the different sites of brain-VBM 2 days after cell seeding.Red box shown central site and carmine box shown peripheral site. The number at the bottow of images show dimension labels of Z-stack. [file 40824_2023_341_MOESM5_ESM.jpg]

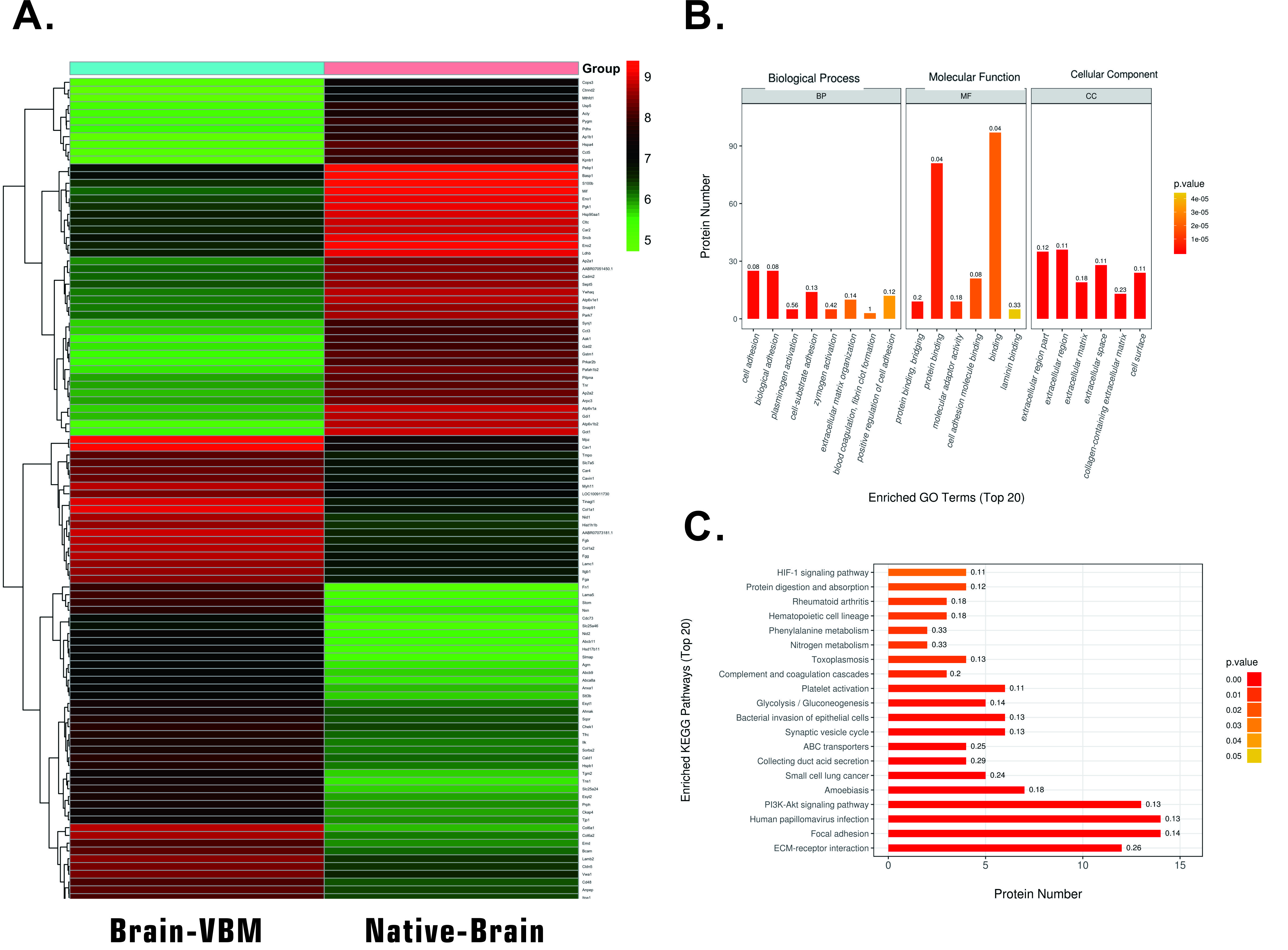

Supplement: Supplementary file 6 — Additional file 6: Figure S3. Bioinformatic analysis of the proteins in brain-VBM and normal brain tissue showed the following: The enriched GO terms (B) showed that the protein functional characteristics were all associated with ECM, cell binding, and cell adhesion. KEGG pathway enrichment(C) showed the 20 cellular signaling pathways with the most differentially expressed proteins. A shows the cluster analysis of differentially expressed proteins. [file 40824_2023_341_MOESM6_ESM.jpg]
